# Analysis of Genes Involved in Body Weight Regulation by Targeted Re-Sequencing

**DOI:** 10.1371/journal.pone.0147904

**Published:** 2016-02-01

**Authors:** Anna-Lena Volckmar, Chung Ting Han, Carolin Pütter, Stefan Haas, Carla I. G. Vogel, Nadja Knoll, Christoph Struve, Maria Göbel, Katharina Haas, Nikolas Herrfurth, Ivonne Jarick, Harald Grallert, Annette Schürmann, Hadi Al-Hasani, Johannes Hebebrand, Sascha Sauer, Anke Hinney

**Affiliations:** 1 Department of Child and Adolescent Psychiatry, Psychosomatics and Psychotherapy, University Hospital Essen, Essen, Germany; 2 Nutrigenomics and Gene Regulation, Max-Planck-Institute for Molecular Genetics, Berlin, Germany; 3 Genomics, Core Facilities and Technology, Institute of Molecular Biology (IMB), Mainz, Germany; 4 Institute for Medical Informatics, Biometry and Epidemiology (IMIBE), University Hospital Essen, Essen, Germany; 5 Department of Animal and Food Production, Santa Catarina State University, Lages, Brazil; 6 Institute of Medical Biometry and Epidemiology, Philipps-University of Marburg, Marburg, Germany; 7 Institute of Epidemiology, Helmholtz-Zentrum Munich, Munich, Germany; 8 Institute of Experimental Diabetology, German Institute of Human Nutrition Potsdam-Rehbruecke, Nuthetal, Germany; 9 German Center for Diabetes Research (DZD), München-Neuherberg, Germany; 10 Institute of Pathobiochemistry, German Diabetes Center and German Center for Diabetes Research, Düsseldorf, Germany; 11 CU Systems Medicine, University of Wuerzburg, Wuerzburg, Germany; GDC, GERMANY

## Abstract

**Introduction:**

Genes involved in body weight regulation that were previously investigated in genome-wide association studies (GWAS) and in animal models were target-enriched followed by massive parallel next generation sequencing.

**Methods:**

We enriched and re-sequenced continuous genomic regions comprising *FTO*, *MC4R*, *TMEM18*, *SDCCAG8*, *TKNS*, *MSRA* and *TBC1D1* in a screening sample of 196 extremely obese children and adolescents with age and sex specific body mass index (BMI) ≥ 99^th^ percentile and 176 lean adults (BMI ≤ 15^th^ percentile). 22 variants were confirmed by Sanger sequencing. Genotyping was performed in up to 705 independent obesity trios (extremely obese child and both parents), 243 extremely obese cases and 261 lean adults.

**Results and Conclusion:**

We detected 20 different non-synonymous variants, one frame shift and one nonsense mutation in the 7 continuous genomic regions in study groups of different weight extremes. For SNP Arg695Cys (rs58983546) in *TBC1D1* we detected nominal association with obesity (p_TDT_ = 0.03 in 705 trios). Eleven of the variants were rare, thus were only detected heterozygously in up to ten individual(s) of the complete screening sample of 372 individuals. Two of them (in *FTO* and *MSRA*) were found in lean individuals, nine in extremely obese. *In silico* analyses of the 11 variants did not reveal functional implications for the mutations. Concordant with our hypothesis we detected a rare variant that potentially leads to loss of FTO function in a lean individual. For *TBC1D1*, in contrary to our hypothesis, the loss of function variant (Arg443Stop) was found in an obese individual. Functional *in vitro* studies are warranted.

## Introduction

Obesity is one of the major health problems, which is associated with increased mortality and morbidity [1]. To date more than 100 body mass index (BMI) associated loci have been published from GWAS [2, 3, 4]. On the other hand, murine models have shown other relevant genes for weight regulation which were not detected by GWAS (e.g. *Tbc1d1* [5]). The aim of this study is to identify functionally relevant mutations in genes involved in body weight regulation derived from either GWAS or murine models. The rationale for the chosen genes is briefly delineated in the following:

### TBC1D1

In SJL mice a specific mutation in the *Tbc1d1* gene (fsAla4047*4119) results in a truncated protein lacking the TBC Rab-GTPase-activating protein domain. The mutation led to resistance to diet-induced obesity [5] and its causality for the phenotype was confirmed in *Tbc1d1* knockout mice [6, 7]. In mouse skeletal muscle cells, knockdown of *Tbc1d1* increased fatty acid uptake and oxidation whereas overexpression of *Tbc1d1* had the opposite effect [5]. Mutations in the human *TBC1D1* gene are associated with increased risk for familial obesity [8, 9]. Polygenic effects on BMI and waist circumference in humans were also described for some *TBC1D1* SNP alleles [10].

### FTO

The fat mass and obesity associated gene (*FTO)* harbors GWAS derived polygenic variants with the largest effect size on BMI (*FTO*; [3, 11]). The body weight of carriers of one risk allele is increased by approximately 1.5 kgs. The effect of the risk alleles in intron 1 of *FTO* on body weight had been replicated in most analyzed study groups, either across the whole life span [3, 11–14] or in all analyzed ethnic groups [11, 15, 16]. *FTO* is highly expressed in the hypothalamus which is the key region for control of food intake [17]. *FTO* belongs to a gene family that is involved in post-translational modifications, DNA repair and fatty acid metabolism [18–20]. *Fto* deficient mice are lean as a consequence of increased energy expenditure [21] and show an improvement in metabolic syndrome in comparison to leptin-deficient mice, wild type or heterozygotes for *Fto* [22]. Mice over expressing *Fto* showed a dose-dependent and diet-independent increase in body weight and fat mass [23]. This is consistent with the finding that obesity associated SNPs in intron 1 increase *FTO* expression in humans [24]. The same SNP in human is associated with increased protein and lower overall energy intake [25].

### TMEM18

The GWAS-derived gene *TMEM18* is associated with increased BMI [11, 13, 26]. Besides obesity, variants in *TMEM18* are associated with earlier onset of menarche [27], metabolic syndrome [28], and increased waist to hip ratio [26]. The expression of Tmem18 in the prefrontal cortex is strongly and positively correlated with body weight in rats [26]. In humans, a methylation dependent elevated expression of *TMEM18* in subcutaneous adipose tissue was associated with BMI and metabolic traits [29]. The transmembrane protein TMEM18 binds DNA with its positively charged C-terminus that contains also a nuclear localization signal and is likely involved in moving the chromatin close to the nuclear membrane [30].

### *SDCCAG8*, *MSRA* and *TNKS*

We [13] previously described variants associated with early-onset extreme obesity in children and adolescents. The loci are located near the serologically defined colon cancer antigen 8 gene (*SDCCAG8*) and between the genes coding for tankyrase (*TNKS*) and for methionine sulfoxide reductase A (*MSRA*). For these three genes, the role in the regulation of weight and energy homeostasis is yet unknown. SDCCAG8 is involved in Bardet-Biedl syndrome; which is a syndromal form of obesity [31, 32]. Variants in *SDCCAG8* led to reduced weight loss in a group of 401 overweight and obese children and adolescents undergoing a lifestyle intervention [13] but had no effect on weight regain after the intervention [33].

TNKS regulates the centrosome function [34] and telomere conservation [35]. Variants in this gene are associated with type 2 diabetes [36] and several cancers (e.g. gastric cancer [37]; breast cancer [38]; colon cancer [39]; lung cancer [40]). MSRA catalyzes the enzymatic reduction of methionine sulfoxide to methionine. The latter is needed to repair oxidative damage of proteins. Obesity is associated with oxidative stress caused by reactive oxygen species (ROS) in the mitochondrion, with chronic excess of ROS that leads to mitochondrial dysfunction in liver and skeletal muscle which contributes to insulin resistance [41]. Mice without Msra show high-fat-diet-induced insulin resistance, most likely due to increased oxidative stress [42]. Variants in MSRA were associated with increased waist circumference and waist-to-hip ratio in a group of 3494 US Hispanic women [43]. Variants in both *TNKS* and *MSRA* did not affect weight loss in the aforementioned weight loss group of 401 overweight and obese children and adolescents [44] and had no effect on weight regain after the intervention [33].

Although heritability of body weight is high [45], GWAS could only detect a small fraction of the assumed heritability. The 97 lead SNPs detected by the currently largest meta-analysis of GWAS for BMI explain only approximately 5% of the total heritability of the variance of BMI [3].

Here, we analyzed the coding region of genes relevant for body weight regulation derived from GWAS (*FTO*, *MC4R*, *SDCCAG8*, *TNKS*/*MSRA*, and *TMEM18*) or animal models (*TBC1D1*) via targeted re-sequencing for potentially causal variants involved in body weight regulation. We used extreme phenotypes (196 individuals with the highest and 176 with the lowest BMI of our previously described case control GWAS dataset; [12, 46]).

## Material and Methods

### Study group

**Fig 1:**
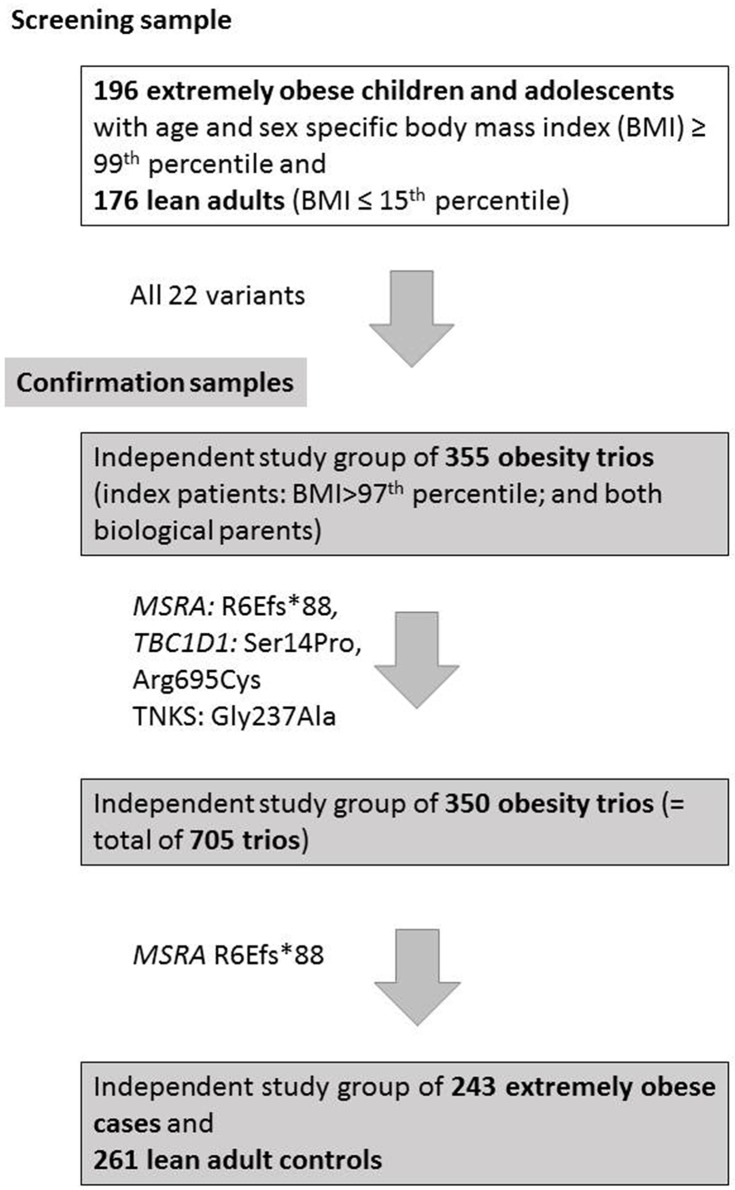
Flowchart of the experimental setup.

The **screening group** consisted of individuals with the highest or lowest BMI, respectively, of our previously described case control GWAS dataset [12, 13]. 196 extremely obese cases (43.9% male, mean age 15.0 years, mean BMI 38.45 kg/m^2^, mean BMI SDS 3.11) and 176 lean controls (52.0% male, mean age 24.74 years, mean BMI 18.15 kg/m^2^, mean BMI SDS -2.31) were analyzed (Fig 1). All individuals were previously analyzed by GWAS (Affymetrix Genome-Wide Human SNP Array 6.0) and were screened by dHPLC for mutations in the *MC4R* [47].

#### Confirmation groups

**(A) Family-based**: A total of 705 obesity trios were used for confirmation. All detected non-synonymous, nonsense or frameshift variants were genotyped in an independent study group of 355 obesity trios (index patients: 45.37% male, mean age 13.72 ± 3.12 years, mean BMI 31.75 ± 6.06 kg/m^2^, mean BMI SDS 4.13 ± 2.06; and both biological parents; subgroup of a study group previously described [48]). The variants rs2279027 (*TBC1D1* [Ser14Pro])), rs58983546 (TBC1D1 [Arg695Cys]), and rs34790717 (TNKS [Gly237Ala]) were additionally genotyped in 350 independent obesity trios (total of 705 trios; see [48]). **(B) Case-control**: The potentially functionally relevant variant *MSRA* [R6Efs*88] was additionally analyzed in 243 young extremely obese cases and 261 lean adult controls by TaqMan assays ([12, 13], Fig 1).

Written informed consent was given by all participants and in case of minors by their parents. The studies were approved by the Ethics Committees of the Universities of Marburg and Duisburg-Essen and were performed in accordance with the Declaration of Helsinki.

### Targeted Re-sequencing by Next-Generation Sequencing (NGS)

5 μg of genomic DNA was sheared to 200 bp by Covaris S2 instrument. 2 μg of sheared DNA was subjected to paired-end library construction, namely end-repair, a-tailing, and amplification with slight modifications [49]. Libraries were individually indexed and pooled prior to targeted enrichment by Agilent SureSelect In-Solution Target Enrichment System. The complete coding regions, including introns, of the genes *FTO*, *MC4R*, *TMEM18*, *SDCCAG8*, *TKNS*, *MSRA* and *TBC1D1* with 10kb 5’ and 3’ flanks to include potential regulatory regions were targeted (S1 Table).

After target enrichment, the libraries were sequenced for 100 bp, using pair-end reads, on an Illumina Genome Analyzer IIx. Read mapping on hg18 and variant calling was performed using the same processing steps as described by Hu et al [50]. In order to reduce false-positive calls only variants with a genotype score >20 were used for subsequent analyses. The raw data were analyzed with pibase (http://www.ikmb.uni-kiel.de/pibase/index.html). For further analyses, the complete exon regions of all protein forming splice variants (http://www.ensembl.org/index.html, hg18) was extracted from the sequencing results. The analysis of 20 lean individuals failed during this process, so that the control group only consisted of 176 individuals.

The *MC4R* was included in the analysis despite being previously screened with an independent method (dHPLC, Sanger re-sequencing, [47]) to serve as a control for the used NGS protocol. Prior to analysis of the *MC4R* variants, the validity threshold for non-synonymous variants was set to a genotype score of ‘20’ ([50], which was a conservative threshold to rather include false positive variants, than to miss an existing mutation. With this threshold, a total of 19 variants were detected in *MC4R* (S2 Table). The NGS results were not corrected for failed samples or low coverage. These 19 variants detected by NGS were compared to the initial results of the dHPLC/Sanger re-sequencing approach. Deviant results were validated with additional Sanger re-sequencing. Of the 19 initial variants (threshold 20), only 5 could be confirmed by the independent methods. All variants that could not be reproduced by Sanger sequencing had a score below ‘100’. Hence, we decided to use a score of ‘100’ as a threshold for analysis of the NGS data for all analyzed genes.

### Variant verification

All detected non-synonymous variants (Table 1) with minor allele frequencies below one percent were uni-directionally Sanger re-sequenced. At least two experienced individuals independently assigned the genotypes; discrepancies were resolved by reaching consensus or by re-sequencing. The primers for the PCRs can be obtained from the authors.

**Table 1:** Number of detected variants per gene (*FTO*, *TMEM18*, *SDCCAG8*, *TKNS*, *MSRA* and *TBC1D1*) in 196 extremely obese children and adolescents and 176 lean adults.

Gene	Length cDNA [kb]	Variants total	Variants per kb	Non synonymous variants	NS variants per kb	InDels	InDels per kb
*FTO*	11,766	6	0.51	3	0.25	0	NA
*MSRA*	1,706	4	2.92	3	2.34	1	0.59
*SDCCAG8*	2,567	6	2.33	2	0.78	0	NA
*TBC1D1*	5,700	17	2.98	11	1.93	0	NA
*TMEM18*	2,762	1	0.36	0	NA	0	NA
*TKNS*	9,620	14	1.46	2	0.21	0	NA

### *In silico* functional analysis

All non-synonymous variants were *in silico* analyzed with MutationTaster (http://www.mutationtaster.org/ [51]), PMUT (http://mmb.pcb.ub.es/PMut/ [52]), PolyPhen-2 (http://genetics.bwh.harvard.edu/pph2/ [53]), and SNAP (https://www.rostlab.org/services/SNAP/ [54]) for potential functional impact on the protein level.

### Genotyping for association analysis

All detected non-synonymous variants were genotyped in 355 obesity trios via MALDI TOF. The variants rs2279027 (*TBC1D1* [Ser14Pro]) and rs58983546 (TBC1D1 [Arg695Cys]) were analyzed by TaqMan Assay (C___1673024_1_ and C__89662374_10, respectively) and the variant rs34790717 (TNKS [Gly237Ala]) by PCR-RFLP (Primers F 3’-GGCAAACGTAAATGCAAAGG-5’ and R 3’-CCTCACCAGAAGACTGGAGG-5’; digest of the C-allele with *Mwo*1) in 350 independent obesity trios. The variant TMP_ESP_8_9912039 (*MSRA* [R6Efs*88]) was additionally genotyped by a custom TaqMan SNP Genotyping Assay (AHZAE78) in 243 extremely obese children and adolescents and 261 lean adult controls.

### Statistical analysis

At the start of the screening we decided to genotype the detected non-synonymous, frameshift and nonsense mutations in 355 independent obesity trios to identify potential transmission disequilibrium [55] for the analyzed variants. At that time, we did not know the allele frequencies of the variants to be detected, hence power calculations could not be performed. The TDT procedure was previously very successful in our hands for infrequent variants (1–3% allele frequency). For instance, we detected significantly reduced transmission of the infrequent allele at the *MC4R* Val103Ile polymorphism to obese children in a relatively small number of obesity trios (n = 520 [56]). We were the first group to describe the polygenic weight lowering effect of the MC4R 103Ile allele; previous case control association studies did not identify the small effect. Hence we regard the trio approach as especially powerful for variants with a low frequency, as stratification effects are mainly eliminated [55]. SNPs with tentatively low p-values in the 355 trios were genotyped in an additional 350 further obesity trios, so that a total of 705 obesity trios was screened for promising SNPs.

Association to obesity was analyzed by standard statistical programs (case-control: Fisher’s exact test from Excel, obesity trios: transmission-disequilibrium test in Excel [55]. All reported p-values are nominal, uncorrected and two-tailed.

## Results

### Screening procedure and quality control

We aimed to identify new mutations by targeted re-sequencing in the continuous genomic regions of seven genes involved in weight regulation. We used a screening sample of 196 obese and 176 lean individuals of different weight extremes; detected variants were confirmed in 355 independent obesity trios, SNPs with a nominal p-value below 0.05 were genotyped in a total of 705 obesity trios (355 plus 350 trios). Additionally, for the variant *MSRA* [R6Efs*88] which likely confers a functional effect, we analyzed 243 young extremely obese cases and 261 lean adult controls ([12, 13], Fig 1) on top of the 705 trios.

*MC4R* was used as a reference gene to define a validity score for the initial discovery of variants derived from re-sequencing of enriched genomic regions (S2 Table). We hence used the genotype score ‘100’ [50] for the remaining genes *TBC1D1*, *FTO*, *TMEM18*, *SDCCAG8*, *TKNS*, and *MSRA* (Table 1).

### Mutational analysis of *TBC1D1*, *FTO*, *TMEM18*, *SDCCAG8*, *TKNS*, and *MSRA*

A total of 49 variants (48 non-synonymous and 1 frameshift, S2 Table) were detected in the sample of 196 extremely obese children and adolescents and 176 lean adults within the 6 genes (*FTO*, *TMEM18*, *SDCCAG8*, *TKNS*, *MSRA* and *TBC1D1*). Of these, 32 variants were known SNPs as listed in the dbSNP. The others were yet not described in the accessible databases (data access for all: November 9^th^ 2015: EVS: http://evs.gs.washington.edu/EVS/, 1000Genomes: http://browser.1000genomes.org/index.html, dbSNP: http://www.ncbi.nlm.nih.gov/snp/, and ExAC: http://exac.broadinstitute.org/variant/). The previously unknown variants had a minor allele frequency below 0.001 and were detected heterozygously in all cases (Table 2). The 22 non-synonymous and frameshift variants detected in the sample were Sanger re-sequenced; only one could not be confirmed (*TNKS* Asn1103Lys).

**Table 2:** Non-synonymous, stop and frameshift variants^1^ detected by NGS of the genes *FTO*, *TMEM18*, *SDCCAG8*, *TKNS*, *MSRA* and *TBC1D1* in 196 extremely obese children and adolescents and 176 lean adults.

				Obese				Lean						
Gene	Variant	Effect on amino acid level	MAF in CEU^2^	11	12	22	pHWG	11	12	22	pHWG	p-value	OR	*In silico* functional prediciton
*FTO*	rs144100465	Cys9Tyr	0.000	196	0	0	1.00	175	1	0	0.97	1	NA	Polymorphism
*FTO*		Val83Leu	0.000	195	1	0	0.97	176	0	0	1.00	1	NA	disease causing
*FTO*	rs145884431	Ala163Thr	0.003	194	2	0	0.94	176	0	0	1.00	0.33	0.22	disease causing
*SDCCAG8*	rs2275155	Glu378Asp	0.275	158	32	6	**0.01**	155	17	4	**0.00**	0.97	0.53	disease causing
*SDCCAG8*	rs79435766	Thr398Met	0.000	195	1	0	0.97	176	0	0	1.00	1	NA	disease causing
*TNKS*	rs34790717	Gly237Ala	0.371	149	44	3	0.90	123	51	2	0.19	0.79	1.36	disease causing
*TNKS*		Pro275Ala	NA	195	1	0	0.97	176	0	0	1.00	1	NA	disease causing
*MSRA*	TMP_ESP_8_9912039	Arg6Glufs*88	0.009	195	1	0	0.97	176	0	0	1.00	1	NA	disease causing
*MSRA*	rs6601444	Thr88Met	0.197	151	43	2	0.58	144	27	5	**0.01**	0.97	0.75	disease causing
*MSRA*		Asp142Tyr	0.000	195	1	0	0.97	176	0	0	1.00	1	NA	Polymorphism
*MSRA*	rs201155438	Gly187Ser	0.000	195	1	0	0.97	176	0	0	1.00	1	NA	Polymorphism
*TBC1D1*	rs2279027	Ser14Pro	0.325	113	65	29	**0.00**	117	54	20	**0.00**	0.18	0.76	disease causing
*TBC1D1*	rs35859249	Arg125Trp	0.139	173	23	0	0.38	157	18	1	0.54	0.775	0.91	disease causing
*TBC1D1*	rs112261209	Arg327Lys	0.012	191	5	0	0.86	173	3	0	0.91	0.596	0.66	disease causing
*TBC1D1*	rs61731607	Ala384Pro	0.042	189	7	0	0.80	174	2	0	0.94	0.148	0.31	disease causing
*TBC1D1*	rs61731610	Gly389Ser	0.041	190	6	0	0.83	174	2	0	0.94	0.220	0.36	disease causing
*TBC1D1*		Arg443*	0.000	195	1	0	0.97	176	0	0	1.00	1	NA	disease causing
*TBC1D1*	rs145177739	Gln619Arg	0.000	195	1	0	0.97	176	0	0	1.00	1	NA	disease causing
*TBC1D1*	rs58983546	Arg695Cys	0.118	168	28	0	0.28	151	24	1	0.97	0.982	0.99	disease causing
*TBC1D1*		Leu838Val	NA	195	1	0	0.97	176	0	0	1.00	1	NA	disease causing	
*TBC1D1*	rs376683121	Arg1091His	0.000	195	1	0	0.97	176	0	0	1.00	1	NA	disease causing	
*TBC1D1*	rs13110318	Arg1136Gln	0.094	191	5	0	0.86	175	1	0	0.97	0.166	0.22	disease causing	

AA: Amino acid; pHWG: p value of the Hardy Weinberg disequilibrium (deviations from HWG are marked in bold); NA: not available; p-value is calculated with Fisher’s exact test; OR: Odd’s Ratio

^1^score >100

^2^ Minor allele frequency (MAF) taken from http://exac.broadinstitute.org/, European cohort.

### Confirmation and genotyping in large independent study groups

Genotyping was performed in up to 705 obesity trios plus 243 young extremely obese cases and 261 lean adult controls [12, 13] independent of the screening sample. All detected non-synonymous, nonsense and frameshift variants were genotyped by MALDI-TOF in 355 independent family-based obesity trios. As most of these variants were rare, nine of the 21 could not be detected in the additional sample. None of the variants showed transmission disequilibrium for obesity in the 355 obesity trios (Table 3).

**Table 3:** Transmission disequilibrium test of the 22 variants detected by NGS in 355 German obesity trios.

				EAF			
Gene	Variant	Effect on amino acid level	Alleles	Index	Parents	OR	p-value
*FTO*	rs144100465	Cys9Tyr	A/**G**	1.15%	1.02%	1.333	0.71
*FTO*		Val83Leu	G/T	0.00%	0.00%	NA	NA
*FTO*	rs145884431	Ala163Thr	A/G	0.85%	0.71%	1.5	0.65
*SDCCAG8*	rs2275155	Glu378Asp	A/**T**	46.46%	44.91%	1.082	0.49
*SDCCAG8*	rs79435766	Thr398Met	C/G	0.00%	0.00%	NA	NA
*TNKS*	rs34790717	Gly237Ala	C/**G**	37.61%	39.16%	0.913	0.46
*TNKS*		Pro275Ala	C/G	0.00%	0.00%	NA	NA
*MSRA*	TMP_ESP_8_9912039	Arg6Glufs*88	CC/—	0.00%	0.00%	NA	NA
*MSRA*	rs6601444	Thr88Met	C/**T**	35.65%	37.76%	1.012	0.57
*MSRA*		Asp142Tyr	G/T	0.00%	0.00%	NA	NA
*MSRA*	rs201155438	Gly187Ser	A/G	0.00%	0.00%	NA	NA
*TBC1D1*	rs2279027	Ser14Pro	A/**G**	48.72%	50.88%	0.6	0.32
*TBC1D1*	rs35859249	Arg125Trp	C/**T**	16.76%	18.92%	1.082	0.66
*TBC1D1*	rs112261209	Arg327Lys	A/**G**	2.27%	1.60%	2.333	0.21
*TBC1D1*	rs61731607	Ala384Pro	C/**G**	6.25%	6.64%	0.704	0.24
*TBC1D1*	rs61731610	Gly389Ser	A/**G**	6.87%	7.25%	0.818	0.53
*TBC1D1*		Arg443*	C/T	0.00%	0.00%	NA	NA
*TBC1D1*	rs145177739	Gln619Arg	A/G	0.00%	0.00%	NA	NA
*TBC1D1*	rs58983546	Arg695Cys	C/**T**	24.86%	23.31%	1.362	0.05
*TBC1D1*		Leu838Val	T/G	0.00%	0.00%	NA	NA
*TBC1D1*	rs376683121	Arg1091His	A/**G**	0.85%	0.71%	1.5	0.65
*TBC1D1*	rs13110318	Arg1136Gln	A/**G**	0.85%	1.16%	0.6	0.48

AA: Amino acid; EAF: Effect allele frequency; NA: not available; bold: Effect allele

Only a few of the detected non-synonymous variants were *in silico* predicted to behave like the wild type protein (*FTO* [Cys9Tyr], *MSRA* [Asp142Tyr] and *MSRA* [Gly187Ser]). For all others, an altered protein function was predicted (Table 4; Mutation Taster).

**Table 4:** Phenotypes of heterozygous carriers of rare non-synonymous variants in the screening sample and extended *in silico* analyses for the respective variants.

Mutation		Gender	Age [years]	BMI [kg/m^2^]	BMI SDS	*In silico* analyses				
*Gene*	Variant					MutationTaster	PMUT	PolyPhen2	SIFT	SNAP
**Obese**										
*FTO*	Ala163Thr	female	17.01	35.86	2.83	disease causing	Pathological 0.7261	Benign 0.042	Tolerated 1.00	Neutral 60%
*FTO*	Ala163Thr	female	16.20	40.28	3.37	disease causing	Pathological 0.7261	Benign 0.042	Tolerated 1.00	Neutral 60%
*SDCCAG8*	Thr398Met	male	14.87	40.94	3.17	disease causing	Neutral 0.1646	Probably damaging 0.998	Tolerated 0.90	Neutral 53%
*TNKS*	Pro275Ala	female	11.54	33.90	3.01	disease causing	Neutral 0.3125	Benign 0.040	Tolerated 1.00	Neutral 85%
*MSRA*	Arg6Glufs*88	female	13.62	42.67	3.51	disease causing	/	/	/	/
*MSRA*	Gly187Ser	male	15.72	40.40	3.16	Polymorphism	Neutral 0.4197	Benign 0.421	Not tolerated 0.01	Non-neutral 58%
*TBC1D1*	Arg443*	female	14.58	34.84	2.92	disease causing	/	/	/	/
*TBC1D1*	Gln619Arg	male	17.15	39.14	3.16	disease causing	Neutral 0.2881	Probably damaging 0.996	Tolerated 0.95	Non-neutral 63%
*TBC1D1*	Leu838Val	male	13.82	35.54	2.85	disease causing	Neutral 0.1036	Probably damaging 1.000	Not tolerated 0.51	Neutral 69%
*TBC1D1*	Arg1091His	male	7.93	41.54	4.15	disease causing	Neutral 0.4649	Benign 0.042	Tolerated 1.00	Neutral 60%
**Lean**										
*FTO*	Cys9Tyr	female	21.78	18.05	-1.62	Polymorphism	Neutral 0.3751	Benign 0.231	Not tolerated 0.45	Neutral 63%
*FTO*	Val83Leu	male	27.75	18.90	-2.40	disease causing	Pathological 0.6685	Probably damaging 0.998	Not tolerated 1.00	Non-neutral 63%
*MSRA*	Asp142Tyr	female	27.78	17.78	-2.19	Polymorphism	Neutral 0.4242	Probably damaging 1.000	Not tolerated 1.00	Non-neutral 87%

#### TBC1D1

Of the 17 variants detected in *TBC1D1*, eleven were non-synonymous. The SNP Arg695Cys (rs58983546) was not associated with obesity in the initial sample (p = 1), but nominally associated in the 355 obesity trios (nominal p = 0.05). We added 350 independent trios and confirmed the association of the minor allele with obesity (nominal p for the complete set of 705 trios = 0.03). The variant is located in a domain of unknown function (DUF3350; PFAM) of TBC1D1; minor allele frequency is 1.25% in CEU (“Utah residents with ancestry from northern and western Europe”, dbSNP).

The stop gain variant *TBC1D1* Arg443* was only detected once in an extremely obese heterozygous carrier (Table 4) out of a total of 551 obese and 419 lean individuals. A mutation leading to a stop codon would result in a loss of the Rab-GTPase-TBC domain which would result in a complete loss of function.

#### FTO

Of the six detected variants in *FTO*, three were rare and non-synonymous (Table 2). These were found in either obese or lean individuals. Interestingly one mutation (Ala163Thr) was detected in two independent obese individuals, implying cryptic relatedness between these mutation carriers [57]. The variant was previously described by Meyre et al. [58] in both obese and lean subjects. The variant is located in a surface loop of FTO but was not tested *in vitro* for functional effects. *In silico* predictions for this variant were variable (Table 4). The other novel FTO variant Val83Leu was detected heterozygously in a lean female. It is located in the substrate recognition lid of the protein and *in silico* methods predict a highly likely functional outcome (Table 4).

#### TMEM18

None of the variants in *TMEM18* altered the amino acid sequence.

#### SDCCAG8

Of the six variants detected in *SDCCAG8*, two were non-synonymous. Both are previously known SNPs (rs2275155 [Glu378Asp], rs79435766 [Thr398Met]). The latter variant was found in an extremely obese male adolescent (Table 4). The variant is located in a coiled coil domain. *In silico* functional prediction for this variant was variable (Table 4).

#### TNKS

In *TNKS*, only 2 of 14 detected variants were non-synonymous. One of them was a previously known SNP (rs34790717 [Gly237Ala]), while the other one was previously unknown (*TNKS* [Pro275Ala]). The rare variant *TNKS* [Pro275Ala] was detected in an extremely obese female. However, *in silico* predictions for this variant mainly implied no functional effect on protein level (Table 4).

#### MSRA

In *MSRA* we identified a frameshift mutation (Arg6Glufs*88; TMP_ESP_8_9912039) in an extremely obese female (age 13.6 years, BMI 42.67 kg/m^2^, 100^th^ age and sex specific BMI percentile, Table 4). Within the 355 obesity trios the variant was detected in an additional unrelated father. This obese carrier (male, age 45 years, BMI 35 kg/m^2^) did not transmit the variant to his extremely obese offspring. We also screened for the frameshift mutation in an independent sample of 243 extremely obese children and adolescents and 261 lean adult controls. Here, the variant was detected in a normal weight adult female (age 26.8 years, BMI 21.3 kg/m^2^) and a lean adult male (age 24.3 years, BMI 16.5 kg/m^2^). Hence, in sum a strong relevance of this variant for body weight regulation is unlikely. Additionally, three non-synonymous variants were detected (*MSRA* rs6601444 [Thr88Met], [Asp142Tyr], and rs201155438 [Gly187Ser]). One of these [187Ser] was found in an extremely obese male adolescent; the other [142Tyr] in a lean participant (Table 4). Both mutations are located in the MSRA domain of the protein. *In silico* predictions for both variants range from neutral to highly functional (Table 4).

## Discussion

We aimed to identify new mutations with relevance for body weight regulation by targeted re-sequencing (NGS) in the coding regions of six genes.

The gene *TBC1D1* which was originally derived from animal studies contained most variants of all analyzed genes (17 total, of these 11 non synonymous, Table 1). SNP Arg695Cys (rs58983546) was not associated with obesity in the initial sample (p = 1), but showed nominal over-transmission of the 695Cys allele in the 355 obesity trios (p = 0.05; Table 3) and an increased sample size of 705 trios (p = 0.03). In the latest GWAS meta-analysis for BMI, the SNP was not associated with obesity (p = 0.38, [3]). We could not confirm the previously described obesity association [8, 9] of variant Arg125Trp (p = 0.66) in 355 obesity trios. However, the allele frequency for the risk allele was higher in our index patients than in a population based CEU sample (MAF in obese 0.17 vs. 0.07 in the ESP “NHLBI Exome Sequencing Project” cohort, dbSNP), or in previous studies (MAF 0.02 in n = 940 [8]; MAF 0.09 in n = 1,109 [9]). Both reported associations referred only to familial extreme obesity; in fact, Meyre et al. [9] were not able to confirm their finding in a population based sample for mild obesity and overweight (n = 4,634). Although the initial positive association was based mainly on females [9], we did not perform sex-stratified analyses as the allele frequency of the variant is too low for sufficiently powered analyses in females only. SNP Ser14Pro (rs2279027) was not associated with obesity in our study groups (Table 3).

All of the rare *TBC1D1* variants (Arg443*, Gln619Arg, Leu838Val, Arg1091His) were detected in obese cases only (see Table 4). The mutation Arg443* was only detected heterozygously in one extremely obese individual. The mutation leads to a loss of the Rab-GTPase-TBC domain and thus presumably to a loss of function. Although Cheng et al. [59] described binding of the Tbc1d1 protein to the adaptor protein APPL1 via N-terminal PTB domains, for signal transduction to partners downstream of TBC1D1, a full Rab-GTPase-TBC domain is necessary. For the other three variants, *in silico* prediction was rather mixed and conservation was not very strong (Gln619Arg: 63% conservation, Leu838Val: 64% conservation, Arg1019His: 71% conservation for 67 analyzed species; http://www.ensembl.org/).

In short, Arg443* is the functionally most relevant variant we detected in *TBC1D1*. Contrary to our hypothesis, this variant was detected in an obese individual. Functional analyses are therefore warranted. *TBC1D1* SNP Arg695Cys (rs58983546) could contribute to the obesity association of the gene. Hence, although the gene was initially identified in mice with diet induced obesity resistance [5] our findings imply that loss of function and function-reducing variants in humans might be relevant for obesity development.

For the most relevant obesity polygene *FTO*, our data are comparable to previous studies [58, 60] as we also detected non-synonymous variants in both obesity and control study groups. The obesity GWAS signals are localized to the first intron of *FTO* [61, 62] and apparently do not affect the amino acid structure of the protein [58] but rather the expression of *FTO* [24]. A recent study indicates that the obesity association of the first intron does not imply *FTO* as the causal gene, but *IRX3* located downstream on the same chromosome which is regulated by the first intron of *FTO* [63]. The FTO variant Val83Leu, detected in a lean adult male, has the highest potential for a functional impact as the *in silico* predictions for this variant all indicate an altered function which would be concordant with the mouse model (lean phenotype in a knock out mouse). However, as the prediction methods can only analyze change of function, this could also hint at an increased function. Hence, *in vitro* analysis of this variant is highly warranted.

For *SDCCAG8*, previous mutation screens focused on obesity in patients with Bardet-Biedl syndrome [32, 64] and revealed frameshift-, nonsense and loss of splicing enhancer mutations. For the genes *SDCCAG8*, *TNKS* and *MSRA*, we expected to find variants leading to a reduced protein function. However, the variants we detected were too infrequent for meaningful obesity association analysis (*SDCCAG8* [Thr398Met], *TNKS* [Pro275Ala], *MSRA* [Arg6GlufsX88], [Asp142Tyr], [Gly187Ser]) or showed no obesity association (*SDCCAG8* [Glu378Asp], *TNKS* [Gly237Ala]; *MSRA* [Thr88Met],). In detail:

(a) The frameshift variant in *MSRA* [Arg6Glufs*88] was initially detected in an extremely obese individual. The functional implications are presumably strong (*in silico* analyses). Only two lean heterozygous mutation carriers were identified in an independent study group (243 extremely obese children and adolescents and 261 lean adult controls). An obese father of the 705 obesity trios did not transmit the variant his extremely obese offspring. Hence, an impact of the mutation on body weight regulation is rather unlikely. (b) The other rare MSRA variant (Gly187Ser) detected in an obese male was mainly predicted to be functionally irrelevant (Table 4). It is located at a position which is not conserved (3% conservation among 68 species, http://www.ensembl.org/). (c) The rare variant Asp142Tyr in *MSRA* was detected in a lean female (Table 4). It is also located in the MSRA domain of the protein. The amino acid position 142 was conserved between 68 species (conservation 71%, http://www.ensembl.org/). Taken together, our findings hint that the detected *MSRA* mutations are unlikely to be relevant for weight regulation.

The two genes *TNKS* and *SDCCAG8*, derived from GWAS meta-analyses in extremely obese children and adolescent, both harbored rare mutations only in the obese cases. The conservative variant Pro275Ala is located in a region of TNKS that is highly conserved (81% in 72 species, http://www.ensembl.org/). Nonetheless *in silico* prediction is mainly neutral (Table 4). Conservation of amino acid position Thr398 is low (41% in 68 species, http://www.ensembl.org/). In sum a strong impact of the detected mutations in *TNKS* and *SDCCAG8* on obesity cannot be derived from our data.

Limitations of our study include a small sample size, thus low power and the lack of functional studies pertaining to the rare non-synonymous and frame shift mutations we discovered. The respective *in vitro* functional studies are highly warranted as the predictive power of *in silico* programs is limited [65]. We did not correct for multiple testing as none of the variants was reproducibly nominally associated with obesity.

In summary, we screened the coding regions of seven genes for variants leading to monogenic forms of obesity. For *FTO*, we detected, concordant with our hypothesis one rare variant leading to a loss of FTO function in a lean individual. In *TBC1D1* discordant to our hypothesis the loss of function variant (Arg443*) was found in an obese individual.

## Supporting Information

S1 Table Genetic regions (hg19/GRCh37) for NGS including the genes (largest transcript) of interest with additional 10kb flank. All analyzed genes (*FTO*, *TMEM18*, *SDCCAG8*, *TKNS*, *MC4R*, *MSRA* and *TBC1D1*) with regions covered.
(DOCX)

S2 Table Comparison of the results of next generation sequencing and Sanger sequencing for the *MC4R* coding region in 196 extremely obese children and adolescents and 176 lean adults. All detected variants in MC4R and the respective scores for quality validation.
(DOCX)

S3 Table List off all variants detected with Score > 100. All variants in the exonic regions of the screened genes *FTO*, *TMEM18*, *SDCCAG8*, *TKNS*, *MC4R*, *MSRA* and *TBC1D1* in 196 extremely obese children and adolescents and 176 lean adults. Every deviant call from wild type is listed in one line including the probability of heterozygousity (column “Zygosity”) and the score for the overall validity of the variant (column “Score”).
(DOCX)
