# Supplementary material for: Analysis of Genes Involved in Body Weight Regulation by Targeted Re-Sequencing
Source: PLoS One. 2016 Feb 1;11(2):e0147904. doi: 10.1371/journal.pone.0147904 (PMC4734691; doi:10.1371/journal.pone.0147904)
Supplement: S1 Table — All analyzed genes (FTO, TMEM18, SDCCAG8, TKNS, MC4R, MSRA and TBC1D1) with regions covered. (DOCX) [file pone.0147904.s001.docx]

**S1 Table: Genetic regions (hg19/GRCh37) for NGS including the genes (largest transcript) of interest with additional 10kb flank**

| **Gene** | **Chr.** | **Region Start** | **Region End** | **Size [bp]** | **Size largest cDNA [bp]** |
| --- | --- | --- | --- | --- | --- |
| *FTO* | 16 | 53,737,875 | 54,158,379 | 534,000 | 11,766 |
| *MC4R* | 18 | 58,028,564 | 58,050,001 | 275,409 | 1,666 |
| *TMEM18* | 2 | 657,973 | 687,439 | 65,800 | 2,762 |
| *SDCCAG8* | 1 | 243,409,307 | 243,673,393 | 234,000 | 2,567 |
| *TNKS / MSRA* | 8 | 9,403,445 | 10,296,401 | 893,000 | 9,620 / 1,706 |
| *TBC1D1* | 4 | 37,882,705 | 38,150,796 | 268,000 | 5,700 |
