# Supplementary material for: Analysis of Genes Involved in Body Weight Regulation by Targeted Re-Sequencing
Source: PLoS One. 2016 Feb 1;11(2):e0147904. doi: 10.1371/journal.pone.0147904 (PMC4734691; doi:10.1371/journal.pone.0147904)
Supplement: S2 Table — All detected variants in MC4R and the respective scores for quality validation. (DOCX) [file pone.0147904.s002.docx]

**S2** **Table: Comparison of the results of next generation sequencing and Sanger sequencing for the *MC4R* coding region in 196 extremely obese children and adolescents and 176 lean adults**

| **Effect on amino acid level** | **rs number or other identifier** | **Number of carriers in NGS** | **NGS Score** | **Sanger** |
| --- | --- | --- | --- | --- |
| Val2= |  | 1 | 43 | 0 |
| Trp15Arg |  | 1 | 28 | 0 |
| **[Tyr35*** | **rs13447324** | **2** | **284-331** | **2** |
| **Asp37Val]** | **rs13447325** | **2** | **263-344** | **2** |
| Leu59Phe |  | 1 | 25 | 0 |
| **Thr101Asn** |  | **1** | **325** | **1** |
| **Val103Ile** | **rs2229616** | **13** | **23-325** | **13** |
| Asp122Glu | COSM1480402 | 1 | 22 | 0 |
| Ser132Pro |  | 1 | 31 | 0 |
| Leu140= |  | 1 | 35 | 0 |
| Arg147= |  | 1 | 22 | 0 |
| Thr178Met | rs140040360 | 1 | 46 | 0 |
| Ile196= |  | 1 | 21 | 0 |
| Pro230Leu | rs13447334 | 1 | 34 | 0 |
| Gly231Asp |  | 1 | 43 | 0 |
| **Ile251Leu** | **rs52820871** | **4** | **116-268** | **4** |
| Val255Ile |  | 1 | 25 | 0 |
| Ser270Pro |  | 1 | 30 | 0 |
| Leu286Met |  | 1 | 34 | 0 |

Variants in bold were confirmed by Sanger re-sequencing.

[haplotype]
