# Supplementary material for: Analysis of Genes Involved in Body Weight Regulation by Targeted Re-Sequencing
Source: PLoS One. 2016 Feb 1;11(2):e0147904. doi: 10.1371/journal.pone.0147904 (PMC4734691; doi:10.1371/journal.pone.0147904)
Supplement: S3 Table — All variants in the exonic regions of the screened genes FTO, TMEM18, SDCCAG8, TKNS, MC4R, MSRA and TBC1D1 in 196 extremely obese children and adolescents and 176 lean adults. Every deviant call from wild type is listed in one line including the probability of heterozygousity (column “Zygosity”) and the score for the overall validity of the variant (column “Score”). (DOCX) [file pone.0147904.s003.docx]

**S3 Table: List off all variants detected with Score > 100.**

| **Gene Location** | **rs number** | **AA exchange** | **WT Allele** | **Zygosity** | **Score** |  |
| --- | --- | --- | --- | --- | --- | --- |
| FTO_Exon1 | rs73609956 | Thr6= | ref=T | hetero=TH_1315318001_P_AG:45 | score=100 |  |
| FTO_Exon1 | rs73609956 | Thr6= | ref=T | hetero=TH_1715022001_P_AG:50 | score=164 |  |
| FTO_Exon3 |  | Val82Leu | ref=G | hetero=TH_418197001_C_AG:37 | score=195 |  |
| FTO_Exon3 |  | Ala162Thr | ref=G | hetero=TH_1315311001_P_AG:40 | score=144 |  |
| FTO_Exon3 |  | Ala162Thr | ref=G | hetero=TH_1815408001_P_AG:48 | score=285 |  |
| FTO_Exon4 |  | Ser256Asn | ref=G | hetero=TH_515046001_P_AG:47 | score=185 |  |
| FTO_Exon4 |  | Ser256Asn | ref=G | hetero=TH_418185001_C_AG:39 | score=168 |  |
| FTO_Exon6 |  | Ile355= | ref=T | hetero=TH_418136001_C_AG:46 | score=243 |  |
| FTO_Exon8 | rs144100465 | Cys9Tyr | ref=G | hetero=TH_418340001_C_AG:40 | score=141 |  |
| FTO_Exon8 | rs2287142 | Lys20= | ref=G | source=TH_1715088001_P_AG: 42 | score=204 |  |
| FTO_Exon8 | rs2287142 | Lys20= | ref=G | hetero=TH_515095001_P_AG:45 | score=329 |  |
| FTO_Exon8 | rs2287142 | Lys20= | ref=G | source=TH_4018046001_C_AG:33 | score=323 |  |
| FTO_Exon8 | rs2287142 | Lys20= | ref=G | source=TH_418141001_C_AG:29 | score=142 |  |
| MC4R_Exon1 | rs52820871 | Ile251Phe | ref=T | hetero=TH_1815396001_P_AG:46 | score=194 |  |
| MC4R_Exon1 | rs52820871 | Ile251Phe | ref=T | hetero=TH_1815408001_P_AG:46 | score=223 |  |
| MC4R_Exon1 | rs52820871 | Ile251Phe | ref=T | hetero=TH_418147001_C_AG:30 | score=116 |  |
| MC4R_Exon1 | rs52820871 | Ile251Phe | ref=T | hetero=TH_418165001_C_AG:50 | score=268 |  |
| MC4R_Exon1 | rs2229616 | Val103Ile | ref=C | hetero=TH_1415112001_P_AG:36 | score=231 |  |
| MC4R_Exon1 | rs2229616 | Val103Ile | ref=C | hetero=TH_1715012001_P_AG:50 | score=210 |  |
| MC4R_Exon1 | rs2229616 | Val103Ile | ref=C | hetero=TH_1715067001_P_AG:32 | score=180 |  |
| MC4R_Exon1 | rs2229616 | Val103Ile | ref=C | hetero=TH_1815293001_P_AG:43 | score=318 |  |
| MC4R_Exon1 | rs2229616 | Val103Ile | ref=C | hetero=TH_1815484001_P_AG:47 | score=312 |  |
| MC4R_Exon1 | rs2229616 | Val103Ile | ref=C | hetero=TH_715071001_P_AG:41 | score=180 |  |
| MC4R_Exon1 | rs2229616 | Val103Ile | ref=C | hetero=TH_4018015001_C_AG:50 | score=125 |  |
| MC4R_Exon1 | rs2229616 | Val103Ile | ref=C | hetero=TH_4018046001_C_AG:46 | score=325 |  |
| MC4R_Exon1 | rs2229616 | Val103Ile | ref=C | hetero=TH_418140001_C_AG:32 | score=138 |  |
| MC4R_Exon1 |  | Thr101Asn | ref=G | hetero=TH_515037001_P_AG:47 | score=325 |  |
| MC4R_Exon1 | rs13447325 | Asp37Val | ref=T | hetero=TH_1815284001_P_AG:41 | score=263 |  |
| MC4R_Exon1 | rs13447325 | Asp37Val | ref=T | hetero=TH_515247001_P_AG:48 | score=344 |  |
| MC4R_Exon1 | rs13447324 | Tyr35Stop | ref=G | hetero=TH_1815284001_P_AG:45 | score=284 |  |
| MC4R_Exon1 | rs13447324 | Tyr35Stop | ref=G | hetero=TH_515247001_P_AG:45 | score=331 |  |
| MSRA_Exon1 |  | Thr5fs*131 | mutations=del | |  |  |
| MSRA_Exon10 |  | Gly187Ser | ref=G | hetero=TH_515095001_P_AG:37 | score=164 |  |
| MSRA_Exon10 | rs3750314 | Gly232= | ref=T | TH_1715022001_P_AG:47 | 272 |  |
| MSRA_Exon10 | rs3750314 | Gly232= | ref=T | TH_1715030001_P_AG:40 | 134 |  |
| MSRA_Exon10 | rs3750314 | Gly232= | ref=T | TH_1715064001_P_AG:43 | 105 |  |
| MSRA_Exon10 | rs3750314 | Gly232= | ref=T | TH_1815221001_P_AG:50 | 100 |  |
| MSRA_Exon10 | rs3750314 | Gly232= | ref=T | TH_515095001_P_AG:43 | 198 |  |
| MSRA_Exon10 | rs3750314 | Gly232= | ref=T | TH_515223001_P_AG:50 | 122 |  |
| MSRA_Exon10 | rs3750314 | Gly232= | ref=T | TH_715095001_P_AG:36 | 134 |  |
| MSRA_Exon10 | rs3750314 | Gly232= | ref=T | TH_715104001_P_AG:37 | 174 |  |
| MSRA_Exon10 | rs3750314 | Gly232= | ref=T | TH_418136001_C_AG:45 | 155 |  |
| MSRA_Exon10 | rs3750314 | Gly232= | ref=T | TH_418142001_C_AG:30 | 104 |  |
| MSRA_Exon10 | rs3750314 | Gly232= | ref=T | TH_418146001_C_AG:42 | 118 |  |
| MSRA_Exon10 | rs3750314 | Gly232= | ref=T | TH_418156001_C_AG:43 | 170 |  |
| MSRA_Exon10 | rs3750314 | Gly232= | ref=T | TH_418165001_C_AG:42 | 147 |  |
| MSRA_Exon10 | rs3750314 | Gly232= | ref=T | TH_418168001_C_AG:28 | 107 |  |
| MSRA_Exon6 |  | Asp142Tyr | ref=G | hetero=TH_418132001_C_AG:50 | score=112 |  |
| MSRA_Exon9 | rs6601444 | Thr88Met | ref=C | hetero=TH_1315249001_P_AG:50 | score=133 |  |
| MSRA_Exon9 | rs6601444 | Thr88Met | ref=C | TH_1315318001_P_AG:45 | 106 |  |
| MSRA_Exon9 | rs6601444 | Thr88Met | ref=C | TH_1315342001_P_AG:45 | 291 |  |
| MSRA_Exon9 | rs6601444 | Thr88Met | ref=C | TH_1415117001_P_AG:26 | 132 |  |
| MSRA_Exon9 | rs6601444 | Thr88Met | ref=C | TH_1415154001_P_AG:29 | 120 |  |
| MSRA_Exon9 | rs6601444 | Thr88Met | ref=C | TH_1715003001_P_AG:45 | 311 |  |
| MSRA_Exon9 | rs6601444 | Thr88Met | ref=C | TH_1715021001_P_AG:45 | 337 |  |
| MSRA_Exon9 | rs6601444 | Thr88Met | ref=C | TH_1715022001_P_AG:44 | 296 |  |
| MSRA_Exon9 | rs6601444 | Thr88Met | ref=C | TH_1715067001_P_AG:30 | 157 |  |
| MSRA_Exon9 | rs6601444 | Thr88Met | ref=C | TH_1715080001_P_AG:34 | 169 |  |
| MSRA_Exon9 | rs6601444 | Thr88Met | ref=C |  | 117 |  |
| MSRA_Exon9 | rs6601444 | Thr88Met | ref=C | TH_1715105001_P_AG:33 | 153 |  |
| MSRA_Exon9 | rs6601444 | Thr88Met | ref=C | TH_1815241001_P_AG:45 | 261 |  |
| MSRA_Exon9 | rs6601444 | Thr88Met | ref=C | TH_1815251001_P_AG:35 | 194 |  |
| MSRA_Exon9 | rs6601444 | Thr88Met | ref=C | TH_1815293001_P_AG:41 | 288 |  |
| MSRA_Exon9 | rs6601444 | Thr88Met | ref=C | TH_1815337001_P_AG:48 | 289 |  |
| MSRA_Exon9 | rs6601444 | Thr88Met | ref=C | TH_515015001_P_AG:45 | 292 |  |
| MSRA_Exon9 | rs6601444 | Thr88Met | ref=C | TH_515018001_P_AG:43 | 218 |  |
| MSRA_Exon9 | rs6601444 | Thr88Met | ref=C | TH_515035001_P_AG:44 | 260 |  |
| MSRA_Exon9 | rs6601444 | Thr88Met | ref=C | TH_515046001_P_AG:40 | 146 |  |
| MSRA_Exon9 | rs6601444 | Thr88Met | ref=C | TH_515055001_P_AG:50 | 264 |  |
| MSRA_Exon9 | rs6601444 | Thr88Met | ref=C | TH_515095001_P_AG:39 | 300 |  |
| MSRA_Exon9 | rs6601444 | Thr88Met | ref=C | TH_515099001_P_AG:31 | 100 |  |
| MSRA_Exon9 | rs6601444 | Thr88Met | ref=C | TH_515112001_P_AG:41 | 261 |  |
| MSRA_Exon9 | rs6601444 | Thr88Met | ref=C | TH_515121001_P_AG:37 | 265 |  |
| MSRA_Exon9 | rs6601444 | Thr88Met | ref=C | TH_515132001_P_AG:50 | 178 |  |
| MSRA_Exon9 | rs6601444 | Thr88Met | ref=C | TH_515151001_P_AG:47 | 256 |  |
| MSRA_Exon9 | rs6601444 | Thr88Met | ref=C | TH_515162001_P_AG:46 | 328 |  |
| MSRA_Exon9 | rs6601444 | Thr88Met | ref=C | TH_515165001_P_AG:47 | 352 |  |
| MSRA_Exon9 | rs6601444 | Thr88Met | ref=C | TH_515180001_P_AG:50 | 133 |  |
| MSRA_Exon9 | rs6601444 | Thr88Met | ref=C |  | 178 |  |
| MSRA_Exon9 | rs6601444 | Thr88Met | ref=C | TH_515206001_P_AG:50 | 220 |  |
| MSRA_Exon9 | rs6601444 | Thr88Met | ref=C | TH_515211001_P_AG:43 | 135 |  |
| MSRA_Exon9 | rs6601444 | Thr88Met | ref=C | TH_515223001_P_AG:47 | 252 |  |
| MSRA_Exon9 | rs6601444 | Thr88Met | ref=C | TH_515244001_P_AG:48 | 260 |  |
| MSRA_Exon9 | rs6601444 | Thr88Met | ref=C | TH_515262001_P_AG:42 | 184 |  |
| MSRA_Exon9 | rs6601444 | Thr88Met | ref=C | TH_515285001_P_AG:50 | 117 |  |
| MSRA_Exon9 | rs6601444 | Thr88Met | ref=C | TH_515294001_P_AG:47 | 330 |  |
| MSRA_Exon9 | rs6601444 | Thr88Met | ref=C | TH_515297001_P_AG:50 | 126 |  |
| MSRA_Exon9 | rs6601444 | Thr88Met | ref=C | TH_515303001_P_AG:43 | 128 |  |
| MSRA_Exon9 | rs6601444 | Thr88Met | ref=C | TH_515333001_P_AG:43 | 182 |  |
| MSRA_Exon9 | rs6601444 | Thr88Met | ref=C | TH_715088001_P_AG:46 | 216 |  |
| MSRA_Exon9 | rs6601444 | Thr88Met | ref=C | TH_715092001_P_AG:47 | 200 |  |
| MSRA_Exon9 | rs6601444 | Thr88Met | ref=C | TH_715184001_P_AG:48 | 317 |  |
| MSRA_Exon9 | rs6601444 | Thr88Met | ref=C | TH_4018004001_C_AG:34 | 129 |  |
| MSRA_Exon9 | rs6601444 | Thr88Met | ref=C |  | 108 |  |
| MSRA_Exon9 | rs6601444 | Thr88Met | ref=C | TH_4018009001_C_AG:40 | 197 |  |
| MSRA_Exon9 | rs6601444 | Thr88Met | ref=C | TH_4018010001_C_AG:38 | 277 |  |
| MSRA_Exon9 | rs6601444 | Thr88Met | ref=C |  | 129 |  |
| MSRA_Exon9 | rs6601444 | Thr88Met | ref=C | TH_4018021001_C_AG:32 | 130 |  |
| MSRA_Exon9 | rs6601444 | Thr88Met | ref=C | TH_4018051001_C_AG:40 | 232 |  |
| MSRA_Exon9 | rs6601444 | Thr88Met | ref=C | TH_4018054001_C_AG:50 | 134 |  |
| MSRA_Exon9 | rs6601444 | Thr88Met | ref=C | TH_4018066001_C_AG:45 | 309 |  |
| MSRA_Exon9 | rs6601444 | Thr88Met | ref=C | TH_418119001_C_AG:39 | 232 |  |
| MSRA_Exon9 | rs6601444 | Thr88Met | ref=C | TH_418121001_C_AG:50 | 263 |  |
| MSRA_Exon9 | rs6601444 | Thr88Met | ref=C | TH_418122001_C_AG:46 | 210 |  |
| MSRA_Exon9 | rs6601444 | Thr88Met | ref=C | TH_418133001_C_AG:33 | 152 |  |
| MSRA_Exon9 | rs6601444 | Thr88Met | ref=C | TH_418135001_C_AG:47 | 183 |  |
| MSRA_Exon9 | rs6601444 | Thr88Met | ref=C | TH_418136001_C_AG:43 | 256 |  |
| MSRA_Exon9 | rs6601444 | Thr88Met | ref=C | TH_418137001_C_AG:45 | 197 |  |
| MSRA_Exon9 | rs6601444 | Thr88Met | ref=C | TH_418156001_C_AG:50 | 195 |  |
| MSRA_Exon9 | rs6601444 | Thr88Met | ref=C | TH_418158001_C_AG:47 | 174 |  |
| MSRA_Exon9 | rs6601444 | Thr88Met | ref=C | TH_418162001_C_AG:41 | 199 |  |
| MSRA_Exon9 | rs6601444 | Thr88Met | ref=C | TH_418165001_C_AG:48 | 280 |  |
| MSRA_Exon9 | rs6601444 | Thr88Met | ref=C | TH_418167001_C_AG:44 | 273 |  |
| MSRA_Exon9 | rs6601444 | Thr88Met | ref=C |  | 138 |  |
| MSRA_Exon9 | rs6601444 | Thr88Met | ref=C | TH_418171001_C_AG:24 | 181 |  |
| MSRA_Exon9 | rs6601444 | Thr88Met | ref=C | TH_418178001_C_AG:50 | 150 |  |
| MSRA_Exon9 | rs6601444 | Thr88Met | ref=C |  | 102 |  |
| MSRA_Exon9 | rs6601444 | Thr88Met | ref=C | TH_418194001_C_AG:33 | 146 |  |
| MSRA_Exon9 | rs6601444 | Thr88Met | ref=C |  | 117 |  |
| MSRA_Exon9 | rs6601444 | Thr88Met | ref=C | TH_418304001_C_AG:42 | 327 |  |
| MSRA_Exon9 | rs6601444 | Thr88Met | ref=C | TH_418306001_C_AG:45 | 230 |  |
| MSRA_Exon9 | rs6601444 | Thr88Met | ref=C | TH_418325001_C_AG:45 | 107 |  |
| MSRA_Exon9 | rs6601444 | Thr88Met | ref=C | TH_418357001_C_AG:44 | 204 |  |
| MSRA_Exon9 | rs6601444 | Thr88Met | ref=C | TH_418712001_C_AG:45 | 260 |  |
| SDCCAG8_Exon11 | rs2275155 | Glu378Asp | ref=A |  | 120 |  |
| SDCCAG8_Exon11 | rs2275155 | Glu378Asp | ref=A | TH_1315376001_P_AG:46 | 147 |  |
| SDCCAG8_Exon11 | rs2275155 | Glu378Asp | ref=A | TH_1715012001_P_AG:34 | 127 |  |
| SDCCAG8_Exon11 | rs2275155 | Glu378Asp | ref=A | TH_1715021001_P_AG:40 | 203 |  |
| SDCCAG8_Exon11 | rs2275155 | Glu378Asp | ref=A | TH_1715022001_P_AG:40 | 214 |  |
| SDCCAG8_Exon11 | rs2275155 | Glu378Asp | ref=A | TH_1715034001_P_AG:45 | 206 |  |
| SDCCAG8_Exon11 | rs2275155 | Glu378Asp | ref=A | TH_1715040001_P_AG:47 | 118 |  |
| SDCCAG8_Exon11 | rs2275155 | Glu378Asp | ref=A | TH_1715053001_P_AG:37 | 152 |  |
| SDCCAG8_Exon11 | rs2275155 | Glu378Asp | ref=A | TH_1715073001_P_AG:44 | 156 |  |
| SDCCAG8_Exon11 | rs2275155 | Glu378Asp | ref=A | TH_1815099001_P_AG:50 | 100 |  |
| SDCCAG8_Exon11 | rs2275155 | Glu378Asp | ref=A | TH_1815247001_P_AG:50 | 243 |  |
| SDCCAG8_Exon11 | rs2275155 | Glu378Asp | ref=A | TH_1815261001_P_AG:48 | 197 |  |
| SDCCAG8_Exon11 | rs2275155 | Glu378Asp | ref=A | TH_1815268001_P_AG:44 | 219 |  |
| SDCCAG8_Exon11 | rs2275155 | Glu378Asp | ref=A |  | 117 |  |
| SDCCAG8_Exon11 | rs2275155 | Glu378Asp | ref=A | TH_1815284001_P_AG:36 | 177 |  |
| SDCCAG8_Exon11 | rs2275155 | Glu378Asp | ref=A | TH_1815285001_P_AG:40 | 142 |  |
| SDCCAG8_Exon11 | rs2275155 | Glu378Asp | ref=A | TH_1815293001_P_AG:43 | 217 |  |
| SDCCAG8_Exon11 | rs2275155 | Glu378Asp | ref=A | TH_1815396001_P_AG:48 | 176 |  |
| SDCCAG8_Exon11 | rs2275155 | Glu378Asp | ref=A | TH_1815484001_P_AG:48 | 201 |  |
| SDCCAG8_Exon11 | rs2275155 | Glu378Asp | ref=A | TH_515015001_P_AG:40 | 158 |  |
| SDCCAG8_Exon11 | rs2275155 | Glu378Asp | ref=A | TH_515037001_P_AG:38 | 132 |  |
| SDCCAG8_Exon11 | rs2275155 | Glu378Asp | ref=A | TH_515046001_P_AG:45 | 100 |  |
| SDCCAG8_Exon11 | rs2275155 | Glu378Asp | ref=A | TH_515080001_P_AG:38 | 124 |  |
| SDCCAG8_Exon11 | rs2275155 | Glu378Asp | ref=A |  | 132 |  |
| SDCCAG8_Exon11 | rs2275155 | Glu378Asp | ref=A | TH_515112001_P_AG:28 | 105 |  |
| SDCCAG8_Exon11 | rs2275155 | Glu378Asp | ref=A | TH_515114001_P_AG:46 | 161 |  |
| SDCCAG8_Exon11 | rs2275155 | Glu378Asp | ref=A | TH_515121001_P_AG:45 | 184 |  |
| SDCCAG8_Exon11 | rs2275155 | Glu378Asp | ref=A | TH_515162001_P_AG:48 | 205 |  |
| SDCCAG8_Exon11 | rs2275155 | Glu378Asp | ref=A |  | 181 |  |
| SDCCAG8_Exon11 | rs2275155 | Glu378Asp | ref=A | TH_515167001_P_AG:33 | 106 |  |
| SDCCAG8_Exon11 | rs2275155 | Glu378Asp | ref=A |  | 126 |  |
| SDCCAG8_Exon11 | rs2275155 | Glu378Asp | ref=A |  | 132 |  |
| SDCCAG8_Exon11 | rs2275155 | Glu378Asp | ref=A | TH_515221001_P_AG:41 | 101 |  |
| SDCCAG8_Exon11 | rs2275155 | Glu378Asp | ref=A | TH_515244001_P_AG:42 | 169 |  |
| SDCCAG8_Exon11 | rs2275155 | Glu378Asp | ref=A | TH_515293001_P_AG:47 | 110 |  |
| SDCCAG8_Exon11 | rs2275155 | Glu378Asp | ref=A | TH_715013001_P_AG:47 | 283 |  |
| SDCCAG8_Exon11 | rs2275155 | Glu378Asp | ref=A | TH_715092001_P_AG:43 | 123 |  |
| SDCCAG8_Exon11 | rs2275155 | Glu378Asp | ref=A | TH_715184001_P_AG:39 | 166 |  |
| SDCCAG8_Exon11 | rs2275155 | Glu378Asp | ref=A | TH_4018027001_C_AG:40 | 275 |  |
| SDCCAG8_Exon11 | rs2275155 | Glu378Asp | ref=A |  | 187 |  |
| SDCCAG8_Exon11 | rs2275155 | Glu378Asp | ref=A | TH_418118001_C_AG:40 | 116 |  |
| SDCCAG8_Exon11 | rs2275155 | Glu378Asp | ref=A |  | 120 |  |
| SDCCAG8_Exon11 | rs2275155 | Glu378Asp | ref=A | TH_418137001_C_AG:47 | 164 |  |
| SDCCAG8_Exon11 | rs2275155 | Glu378Asp | ref=A | TH_418142001_C_AG:37 | 107 |  |
| SDCCAG8_Exon11 | rs2275155 | Glu378Asp | ref=A | TH_418147001_C_AG:46 | 136 |  |
| SDCCAG8_Exon11 | rs2275155 | Glu378Asp | ref=A | TH_418148001_C_AG:30 | 133 |  |
| SDCCAG8_Exon11 | rs2275155 | Glu378Asp | ref=A | TH_418156001_C_AG:35 | 113 |  |
| SDCCAG8_Exon11 | rs2275155 | Glu378Asp | ref=A | TH_418166001_C_AG:43 | 107 |  |
| SDCCAG8_Exon11 | rs2275155 | Glu378Asp | ref=A | TH_418171001_C_AG:48 | 225 |  |
| SDCCAG8_Exon11 | rs2275155 | Glu378Asp | ref=A | TH_418182001_C_AG:37 | 122 |  |
| SDCCAG8_Exon11 | rs2275155 | Glu378Asp | ref=A | TH_418194001_C_AG:50 | 107 |  |
| SDCCAG8_Exon11 | rs2275155 | Glu378Asp | ref=A | TH_418302001_C_AG:31 | 100 |  |
| SDCCAG8_Exon11 | rs2275155 | Glu378Asp | ref=A |  | 181 |  |
| SDCCAG8_Exon11 | rs2275155 | Glu378Asp | ref=A | TH_418319001_C_AG:50 | 115 |  |
| SDCCAG8_Exon11 | rs2275155 | Glu378Asp | ref=A | TH_418357001_C_AG:37 | 100 |  |
| SDCCAG8_Exon11 | rs2275155 | Glu378Asp | ref=A | TH_418361001_C_AG:43 | 131 |  |
| SDCCAG8_Exon11 | rs2275155 | Glu378Asp | ref=A |  | 111 |  |
| SDCCAG8_Exon11 | rs2275155 | Glu378Asp | ref=A | TH_418701001_C_AG:50 | 194 |  |
| SDCCAG8_Exon11 | rs2275155 | Glu378Asp | ref=A | TH_418739001_C_AG:45 | 135 |  |
| SDCCAG8_Exon11 | rs79435766 | Thr398Met | ref=C | hetero=TH_515195001_P_AG:45 | score=252 |  |
| SDCCAG8_Exon15 | rs10927011 | Glu575= | ref=G | TH_1315304001_P_AG:45 | 187 |  |
| SDCCAG8_Exon15 | rs10927011 | Glu575= | ref=G | TH_1315332001_P_AG:42 | 146 |  |
| SDCCAG8_Exon15 | rs10927011 | Glu575= | ref=G |  | 105 |  |
| SDCCAG8_Exon15 | rs10927011 | Glu575= | ref=G | TH_1415115001_P_AG:42 | 148 |  |
| SDCCAG8_Exon15 | rs10927011 | Glu575= | ref=G | TH_1415121001_P_AG:45 | 250 |  |
| SDCCAG8_Exon15 | rs10927011 | Glu575= | ref=G | TH_1415145001_P_AG:38 | 277 |  |
| SDCCAG8_Exon15 | rs10927011 | Glu575= | ref=G | TH_1715003001_P_AG:41 | 180 |  |
| SDCCAG8_Exon15 | rs10927011 | Glu575= | ref=G |  | 111 |  |
| SDCCAG8_Exon15 | rs10927011 | Glu575= | ref=G |  | 102 |  |
| SDCCAG8_Exon15 | rs10927011 | Glu575= | ref=G | TH_1715022001_P_AG:50 | 260 |  |
| SDCCAG8_Exon15 | rs10927011 | Glu575= | ref=G |  | 108 |  |
| SDCCAG8_Exon15 | rs10927011 | Glu575= | ref=G | TH_1715034001_P_AG:37 | 259 |  |
| SDCCAG8_Exon15 | rs10927011 | Glu575= | ref=G |  | 147 |  |
| SDCCAG8_Exon15 | rs10927011 | Glu575= | ref=G | TH_1715050001_P_AG:45 | 247 |  |
| SDCCAG8_Exon15 | rs10927011 | Glu575= | ref=G | TH_1715051001_P_AG:38 | 164 |  |
| SDCCAG8_Exon15 | rs10927011 | Glu575= | ref=G | TH_1715053001_P_AG:32 | 164 |  |
| SDCCAG8_Exon15 | rs10927011 | Glu575= | ref=G | TH_1715065001_P_AG:41 | 120 |  |
| SDCCAG8_Exon15 | rs10927011 | Glu575= | ref=G | TH_1715067001_P_AG:43 | 119 |  |
| SDCCAG8_Exon15 | rs10927011 | Glu575= | ref=G | TH_1715073001_P_AG:39 | 153 |  |
| SDCCAG8_Exon15 | rs10927011 | Glu575= | ref=G | TH_1715093001_P_AG:42 | 139 |  |
| SDCCAG8_Exon15 | rs10927011 | Glu575= | ref=G | TH_1715099001_P_AG:42 | 125 |  |
| SDCCAG8_Exon15 | rs10927011 | Glu575= | ref=G | TH_1715105001_P_AG:47 | 162 |  |
| SDCCAG8_Exon15 | rs10927011 | Glu575= | ref=G |  | 108 |  |
| SDCCAG8_Exon15 | rs10927011 | Glu575= | ref=G | TH_1815247001_P_AG:47 | 260 |  |
| SDCCAG8_Exon15 | rs10927011 | Glu575= | ref=G | TH_1815251001_P_AG:48 | 192 |  |
| SDCCAG8_Exon15 | rs10927011 | Glu575= | ref=G |  | 144 |  |
| SDCCAG8_Exon15 | rs10927011 | Glu575= | ref=G | TH_1815284001_P_AG:47 | 273 |  |
| SDCCAG8_Exon15 | rs10927011 | Glu575= | ref=G | TH_1815285001_P_AG:33 | 103 |  |
| SDCCAG8_Exon15 | rs10927011 | Glu575= | ref=G | TH_1815349001_P_AG:46 | 204 |  |
| SDCCAG8_Exon15 | rs10927011 | Glu575= | ref=G | TH_1815368001_P_AG:50 | 131 |  |
| SDCCAG8_Exon15 | rs10927011 | Glu575= | ref=G | TH_1815396001_P_AG:48 | 224 |  |
| SDCCAG8_Exon15 | rs10927011 | Glu575= | ref=G | TH_515037001_P_AG:47 | 271 |  |
| SDCCAG8_Exon15 | rs10927011 | Glu575= | ref=G | TH_515062001_P_AG:46 | 215 |  |
| SDCCAG8_Exon15 | rs10927011 | Glu575= | ref=G | TH_515073001_P_AG:50 | 163 |  |
| SDCCAG8_Exon15 | rs10927011 | Glu575= | ref=G |  | 126 |  |
| SDCCAG8_Exon15 | rs10927011 | Glu575= | ref=G | TH_515080001_P_AG:35 | 108 |  |
| SDCCAG8_Exon15 | rs10927011 | Glu575= | ref=G |  | 108 |  |
| SDCCAG8_Exon15 | rs10927011 | Glu575= | ref=G | TH_515106001_P_AG:39 | 170 |  |
| SDCCAG8_Exon15 | rs10927011 | Glu575= | ref=G | TH_515112001_P_AG:50 | 214 |  |
| SDCCAG8_Exon15 | rs10927011 | Glu575= | ref=G | TH_515121001_P_AG:50 | 247 |  |
| SDCCAG8_Exon15 | rs10927011 | Glu575= | ref=G | TH_515162001_P_AG:46 | 245 |  |
| SDCCAG8_Exon15 | rs10927011 | Glu575= | ref=G | TH_515164001_P_AG:47 | 260 |  |
| SDCCAG8_Exon15 | rs10927011 | Glu575= | ref=G | TH_515180001_P_AG:46 | 123 |  |
| SDCCAG8_Exon15 | rs10927011 | Glu575= | ref=G | TH_515185001_P_AG:46 | 125 |  |
| SDCCAG8_Exon15 | rs10927011 | Glu575= | ref=G | TH_515186001_P_AG:48 | 223 |  |
| SDCCAG8_Exon15 | rs10927011 | Glu575= | ref=G |  | 150 |  |
| SDCCAG8_Exon15 | rs10927011 | Glu575= | ref=G | TH_515206001_P_AG:36 | 159 |  |
| SDCCAG8_Exon15 | rs10927011 | Glu575= | ref=G | TH_515221001_P_AG:45 | 250 |  |
| SDCCAG8_Exon15 | rs10927011 | Glu575= | ref=G |  | 102 |  |
| SDCCAG8_Exon15 | rs10927011 | Glu575= | ref=G | TH_515244001_P_AG:36 | 144 |  |
| SDCCAG8_Exon15 | rs10927011 | Glu575= | ref=G |  | 223 |  |
| SDCCAG8_Exon15 | rs10927011 | Glu575= | ref=G | TH_515280001_P_AG:41 | 104 |  |
| SDCCAG8_Exon15 | rs10927011 | Glu575= | ref=G |  | 162 |  |
| SDCCAG8_Exon15 | rs10927011 | Glu575= | ref=G | TH_515308001_P_AG:45 | 101 |  |
| SDCCAG8_Exon15 | rs10927011 | Glu575= | ref=G | TH_715006001_P_AG:37 | 113 |  |
| SDCCAG8_Exon15 | rs10927011 | Glu575= | ref=G | TH_715063001_P_AG:47 | 168 |  |
| SDCCAG8_Exon15 | rs10927011 | Glu575= | ref=G | TH_715064001_P_AG:40 | 180 |  |
| SDCCAG8_Exon15 | rs10927011 | Glu575= | ref=G | TH_715069001_P_AG:46 | 149 |  |
| SDCCAG8_Exon15 | rs10927011 | Glu575= | ref=G | TH_715071001_P_AG:42 | 142 |  |
| SDCCAG8_Exon15 | rs10927011 | Glu575= | ref=G | TH_715087001_P_AG:37 | 161 |  |
| SDCCAG8_Exon15 | rs10927011 | Glu575= | ref=G | TH_715184001_P_AG:41 | 229 |  |
| SDCCAG8_Exon15 | rs10927011 | Glu575= | ref=G | TH_4018006001_C_AG:42 | 133 |  |
| SDCCAG8_Exon15 | rs10927011 | Glu575= | ref=G | TH_4018009001_C_AG:48 | 210 |  |
| SDCCAG8_Exon15 | rs10927011 | Glu575= | ref=G |  | 111 |  |
| SDCCAG8_Exon15 | rs10927011 | Glu575= | ref=G | TH_4018024001_C_AG:44 | 265 |  |
| SDCCAG8_Exon15 | rs10927011 | Glu575= | ref=G | TH_4018027001_C_AG:41 | 300 |  |
| SDCCAG8_Exon15 | rs10927011 | Glu575= | ref=G | TH_4018059001_C_AG:50 | 132 |  |
| SDCCAG8_Exon15 | rs10927011 | Glu575= | ref=G | TH_4018066001_C_AG:46 | 314 |  |
| SDCCAG8_Exon15 | rs10927011 | Glu575= | ref=G | TH_418117001_C_AG:48 | 219 |  |
| SDCCAG8_Exon15 | rs10927011 | Glu575= | ref=G | TH_418118001_C_AG:50 | 108 |  |
| SDCCAG8_Exon15 | rs10927011 | Glu575= | ref=G | TH_418121001_C_AG:45 | 198 |  |
| SDCCAG8_Exon15 | rs10927011 | Glu575= | ref=G | TH_418122001_C_AG:50 | 143 |  |
| SDCCAG8_Exon15 | rs10927011 | Glu575= | ref=G |  | 129 |  |
| SDCCAG8_Exon15 | rs10927011 | Glu575= | ref=G | TH_418124001_C_AG:32 | 129 |  |
| SDCCAG8_Exon15 | rs10927011 | Glu575= | ref=G | TH_418125001_C_AG:42 | 242 |  |
| SDCCAG8_Exon15 | rs10927011 | Glu575= | ref=G | TH_418127001_C_AG:48 | 254 |  |
| SDCCAG8_Exon15 | rs10927011 | Glu575= | ref=G | TH_418128001_C_AG:41 | 207 |  |
| SDCCAG8_Exon15 | rs10927011 | Glu575= | ref=G | TH_418134001_C_AG:41 | 103 |  |
| SDCCAG8_Exon15 | rs10927011 | Glu575= | ref=G | TH_418136001_C_AG:40 | 152 |  |
| SDCCAG8_Exon15 | rs10927011 | Glu575= | ref=G | TH_418137001_C_AG:42 | 168 |  |
| SDCCAG8_Exon15 | rs10927011 | Glu575= | ref=G | TH_418139001_C_AG:47 | 161 |  |
| SDCCAG8_Exon15 | rs10927011 | Glu575= | ref=G | TH_418142001_C_AG:37 | 153 |  |
| SDCCAG8_Exon15 | rs10927011 | Glu575= | ref=G | TH_418144001_C_AG:46 | 126 |  |
| SDCCAG8_Exon15 | rs10927011 | Glu575= | ref=G | TH_418146001_C_AG:47 | 148 |  |
| SDCCAG8_Exon15 | rs10927011 | Glu575= | ref=G | TH_418148001_C_AG:50 | 270 |  |
| SDCCAG8_Exon15 | rs10927011 | Glu575= | ref=G | TH_418156001_C_AG:40 | 158 |  |
| SDCCAG8_Exon15 | rs10927011 | Glu575= | ref=G | TH_418159001_C_AG:42 | 151 |  |
| SDCCAG8_Exon15 | rs10927011 | Glu575= | ref=G | TH_418160001_C_AG:47 | 156 |  |
| SDCCAG8_Exon15 | rs10927011 | Glu575= | ref=G | TH_418165001_C_AG:45 | 227 |  |
| SDCCAG8_Exon15 | rs10927011 | Glu575= | ref=G | TH_418167001_C_AG:33 | 151 |  |
| SDCCAG8_Exon15 | rs10927011 | Glu575= | ref=G | TH_418169001_C_AG:45 | 178 |  |
| SDCCAG8_Exon15 | rs10927011 | Glu575= | ref=G | TH_418171001_C_AG:28 | 164 |  |
| SDCCAG8_Exon15 | rs10927011 | Glu575= | ref=G | TH_418178001_C_AG:28 | 126 |  |
| SDCCAG8_Exon15 | rs10927011 | Glu575= | ref=G | TH_418181001_C_AG:46 | 131 |  |
| SDCCAG8_Exon15 | rs10927011 | Glu575= | ref=G | TH_418182001_C_AG:33 | 113 |  |
| SDCCAG8_Exon15 | rs10927011 | Glu575= | ref=G | TH_418185001_C_AG:38 | 140 |  |
| SDCCAG8_Exon15 | rs10927011 | Glu575= | ref=G | TH_418195001_C_AG:43 | 140 |  |
| SDCCAG8_Exon15 | rs10927011 | Glu575= | ref=G | TH_418197001_C_AG:50 | 239 |  |
| SDCCAG8_Exon15 | rs10927011 | Glu575= | ref=G | TH_418300001_C_AG:40 | 246 |  |
| SDCCAG8_Exon15 | rs10927011 | Glu575= | ref=G | TH_418306001_C_AG:35 | 113 |  |
| SDCCAG8_Exon15 | rs10927011 | Glu575= | ref=G | TH_418352001_C_AG:50 | 195 |  |
| SDCCAG8_Exon15 | rs10927011 | Glu575= | ref=G | TH_418361001_C_AG:50 | 212 |  |
| SDCCAG8_Exon15 | rs10927011 | Glu575= | ref=G |  | 123 |  |
| SDCCAG8_Exon15 | rs10927011 | Glu575= | ref=G | TH_418698001_C_AG:43 | 129 |  |
| SDCCAG8_Exon15 | rs10927011 | Glu575= | ref=G | TH_418701001_C_AG:45 | 217 |  |
| SDCCAG8_Exon15 | rs10927011 | Glu575= | ref=G | TH_418739001_C_AG:50 | 200 |  |
| SDCCAG8_Exon15 | rs10927011 | Glu575= | ref=G | TH_418748001_C_AG:44 | 289 |  |
| SDCCAG8_Exon16 |  | Thr605= | ref=A | hetero=TH_515095001_P_AG:50 | score=356 |  |
| SDCCAG8_Exon3 |  | Ser89= | ref=T | hetero=TH_418170001_C_AG:42 | score=228 |  |
| SDCCAG8_Exon8 | rs976529 | Thr304= | ref=C | hetero=TH_1315347001_P_AG:47 | score=263 |  |
| SDCCAG8_Exon8 | rs976529 | Thr304= | ref=C | hetero=TH_1715093001_P_AG:50 | score=232 |  |
| SDCCAG8_Exon8 | rs976529 | Thr304= | ref=C | hetero=TH_1815408001_P_AG:48 | score=330 |  |
| SDCCAG8_Exon8 | rs976529 | Thr304= | ref=C | hetero=TH_515106001_P_AG:40 | score=294 |  |
| SDCCAG8_Exon8 | rs976529 | Thr304= | ref=C | hetero=TH_515213001_P_AG:43 | score=303 |  |
| SDCCAG8_Exon8 | rs976529 | Thr304= | ref=C | hetero=TH_4018036001_C_AG:38 | score=112 |  |
| SDCCAG8_Exon8 | rs976529 | Thr304= | ref=C | hetero=TH_418147001_C_AG:31 | score=109 |  |
| SDCCAG8_Exon8 | rs976529 | Thr304= | ref=C | hetero=TH_418161001_C_AG:50 | score=112 |  |
| SDCCAG8_Exon8 | rs976529 | Thr304= | ref=C | hetero=TH_418164001_C_AG:34 | score=161 |  |
| SDCCAG8_Exon8 | rs976529 | Thr304= | ref=C | hetero=TH_418344001_C_AG:47 | score=166 |  |
| SDCCAG8_Exon8 | rs976529 | Thr304= | ref=C | hetero=TH_418358001_C_AG:44 | score=252 |  |
| TBC1D1_Exon1 | rs2279027 | Ser14Pro | ref=T |  | 114 |  |
| TBC1D1_Exon1 | rs2279027 | Ser14Pro | ref=T | TH_1315249001_P_AG:36 | 126 |  |
| TBC1D1_Exon1 | rs2279027 | Ser14Pro | ref=T | TH_1315342001_P_AG:33 | 171 |  |
| TBC1D1_Exon1 | rs2279027 | Ser14Pro | ref=T |  | 105 |  |
| TBC1D1_Exon1 | rs2279027 | Ser14Pro | ref=T |  | 120 |  |
| TBC1D1_Exon1 | rs2279027 | Ser14Pro | ref=T | TH_1415117001_P_AG:45 | 256 |  |
| TBC1D1_Exon1 | rs2279027 | Ser14Pro | ref=T |  | 138 |  |
| TBC1D1_Exon1 | rs2279027 | Ser14Pro | ref=T |  | 135 |  |
| TBC1D1_Exon1 | rs2279027 | Ser14Pro | ref=T | TH_1415145001_P_AG:48 | 349 |  |
| TBC1D1_Exon1 | rs2279027 | Ser14Pro | ref=T | TH_1415146001_P_AG:35 | 119 |  |
| TBC1D1_Exon1 | rs2279027 | Ser14Pro | ref=T | TH_1415154001_P_AG:41 | 132 |  |
| TBC1D1_Exon1 | rs2279027 | Ser14Pro | ref=T | TH_1715003001_P_AG:48 | 330 |  |
| TBC1D1_Exon1 | rs2279027 | Ser14Pro | ref=T |  | 129 |  |
| TBC1D1_Exon1 | rs2279027 | Ser14Pro | ref=T | TH_1715012001_P_AG:40 | 222 |  |
| TBC1D1_Exon1 | rs2279027 | Ser14Pro | ref=T |  | 223 |  |
| TBC1D1_Exon1 | rs2279027 | Ser14Pro | ref=T |  | 226 |  |
| TBC1D1_Exon1 | rs2279027 | Ser14Pro | ref=T |  | 144 |  |
| TBC1D1_Exon1 | rs2279027 | Ser14Pro | ref=T |  | 102 |  |
| TBC1D1_Exon1 | rs2279027 | Ser14Pro | ref=T |  | 211 |  |
| TBC1D1_Exon1 | rs2279027 | Ser14Pro | ref=T |  | 223 |  |
| TBC1D1_Exon1 | rs2279027 | Ser14Pro | ref=T |  | 150 |  |
| TBC1D1_Exon1 | rs2279027 | Ser14Pro | ref=T | TH_1715053001_P_AG:47 | 276 |  |
| TBC1D1_Exon1 | rs2279027 | Ser14Pro | ref=T | TH_1715067001_P_AG:46 | 244 |  |
| TBC1D1_Exon1 | rs2279027 | Ser14Pro | ref=T | TH_1715073001_P_AG:43 | 176 |  |
| TBC1D1_Exon1 | rs2279027 | Ser14Pro | ref=T |  | 109 |  |
| TBC1D1_Exon1 | rs2279027 | Ser14Pro | ref=T | TH_1715087001_P_AG:34 | 169 |  |
| TBC1D1_Exon1 | rs2279027 | Ser14Pro | ref=T | TH_1715090001_P_AG:43 | 146 |  |
| TBC1D1_Exon1 | rs2279027 | Ser14Pro | ref=T | TH_1815079001_P_AG:46 | 269 |  |
| TBC1D1_Exon1 | rs2279027 | Ser14Pro | ref=T | TH_1815099001_P_AG:38 | 233 |  |
| TBC1D1_Exon1 | rs2279027 | Ser14Pro | ref=T | TH_1815131001_P_AG:44 | 258 |  |
| TBC1D1_Exon1 | rs2279027 | Ser14Pro | ref=T | TH_1815247001_P_AG:46 | 277 |  |
| TBC1D1_Exon1 | rs2279027 | Ser14Pro | ref=T |  | 147 |  |
| TBC1D1_Exon1 | rs2279027 | Ser14Pro | ref=T | TH_1815261001_P_AG:43 | 241 |  |
| TBC1D1_Exon1 | rs2279027 | Ser14Pro | ref=T | TH_1815268001_P_AG:46 | 300 |  |
| TBC1D1_Exon1 | rs2279027 | Ser14Pro | ref=T | TH_1815272001_P_AG:41 | 169 |  |
| TBC1D1_Exon1 | rs2279027 | Ser14Pro | ref=T |  | 152 |  |
| TBC1D1_Exon1 | rs2279027 | Ser14Pro | ref=T | TH_1815313001_P_AG:39 | 192 |  |
| TBC1D1_Exon1 | rs2279027 | Ser14Pro | ref=T | TH_1815349001_P_AG:47 | 228 |  |
| TBC1D1_Exon1 | rs2279027 | Ser14Pro | ref=T | TH_1815396001_P_AG:45 | 246 |  |
| TBC1D1_Exon1 | rs2279027 | Ser14Pro | ref=T | TH_1815408001_P_AG:47 | 265 |  |
| TBC1D1_Exon1 | rs2279027 | Ser14Pro | ref=T | TH_1815443001_P_AG:47 | 162 |  |
| TBC1D1_Exon1 | rs2279027 | Ser14Pro | ref=T | TH_1815484001_P_AG:46 | 319 |  |
| TBC1D1_Exon1 | rs2279027 | Ser14Pro | ref=T |  | 117 |  |
| TBC1D1_Exon1 | rs2279027 | Ser14Pro | ref=T | TH_515015001_P_AG:42 | 241 |  |
| TBC1D1_Exon1 | rs2279027 | Ser14Pro | ref=T | TH_515016001_P_AG:40 | 149 |  |
| TBC1D1_Exon1 | rs2279027 | Ser14Pro | ref=T | TH_515018001_P_AG:45 | 246 |  |
| TBC1D1_Exon1 | rs2279027 | Ser14Pro | ref=T | TH_515035001_P_AG:40 | 209 |  |
| TBC1D1_Exon1 | rs2279027 | Ser14Pro | ref=T | TH_515037001_P_AG:43 | 304 |  |
| TBC1D1_Exon1 | rs2279027 | Ser14Pro | ref=T | TH_515039001_P_AG:43 | 293 |  |
| TBC1D1_Exon1 | rs2279027 | Ser14Pro | ref=T | TH_515055001_P_AG:37 | 174 |  |
| TBC1D1_Exon1 | rs2279027 | Ser14Pro | ref=T | TH_515062001_P_AG:38 | 148 |  |
| TBC1D1_Exon1 | rs2279027 | Ser14Pro | ref=T | TH_515074001_P_AG:37 | 242 |  |
| TBC1D1_Exon1 | rs2279027 | Ser14Pro | ref=T |  | 162 |  |
| TBC1D1_Exon1 | rs2279027 | Ser14Pro | ref=T | TH_515080001_P_AG:36 | 123 |  |
| TBC1D1_Exon1 | rs2279027 | Ser14Pro | ref=T |  | 143 |  |
| TBC1D1_Exon1 | rs2279027 | Ser14Pro | ref=T |  | 205 |  |
| TBC1D1_Exon1 | rs2279027 | Ser14Pro | ref=T | TH_515106001_P_AG:31 | 193 |  |
| TBC1D1_Exon1 | rs2279027 | Ser14Pro | ref=T |  | 150 |  |
| TBC1D1_Exon1 | rs2279027 | Ser14Pro | ref=T | TH_515121001_P_AG:49 | 334 |  |
| TBC1D1_Exon1 | rs2279027 | Ser14Pro | ref=T |  | 108 |  |
| TBC1D1_Exon1 | rs2279027 | Ser14Pro | ref=T | TH_515151001_P_AG:37 | 218 |  |
| TBC1D1_Exon1 | rs2279027 | Ser14Pro | ref=T | TH_515162001_P_AG:45 | 302 |  |
| TBC1D1_Exon1 | rs2279027 | Ser14Pro | ref=T | TH_515164001_P_AG:46 | 316 |  |
| TBC1D1_Exon1 | rs2279027 | Ser14Pro | ref=T | TH_515178001_P_AG:33 | 128 |  |
| TBC1D1_Exon1 | rs2279027 | Ser14Pro | ref=T | TH_515188001_P_AG:42 | 249 |  |
| TBC1D1_Exon1 | rs2279027 | Ser14Pro | ref=T |  | 174 |  |
| TBC1D1_Exon1 | rs2279027 | Ser14Pro | ref=T | TH_515201001_P_AG:45 | 186 |  |
| TBC1D1_Exon1 | rs2279027 | Ser14Pro | ref=T |  | 144 |  |
| TBC1D1_Exon1 | rs2279027 | Ser14Pro | ref=T |  | 111 |  |
| TBC1D1_Exon1 | rs2279027 | Ser14Pro | ref=T | TH_515213001_P_AG:40 | 163 |  |
| TBC1D1_Exon1 | rs2279027 | Ser14Pro | ref=T | TH_515215001_P_AG:42 | 309 |  |
| TBC1D1_Exon1 | rs2279027 | Ser14Pro | ref=T | TH_515221001_P_AG:45 | 290 |  |
| TBC1D1_Exon1 | rs2279027 | Ser14Pro | ref=T | TH_515223001_P_AG:48 | 287 |  |
| TBC1D1_Exon1 | rs2279027 | Ser14Pro | ref=T |  | 153 |  |
| TBC1D1_Exon1 | rs2279027 | Ser14Pro | ref=T | TH_515247001_P_AG:45 | 343 |  |
| TBC1D1_Exon1 | rs2279027 | Ser14Pro | ref=T | TH_515280001_P_AG:43 | 183 |  |
| TBC1D1_Exon1 | rs2279027 | Ser14Pro | ref=T | TH_515294001_P_AG:45 | 333 |  |
| TBC1D1_Exon1 | rs2279027 | Ser14Pro | ref=T | TH_515308001_P_AG:46 | 136 |  |
| TBC1D1_Exon1 | rs2279027 | Ser14Pro | ref=T | TH_715063001_P_AG:37 | 115 |  |
| TBC1D1_Exon1 | rs2279027 | Ser14Pro | ref=T | TH_715064001_P_AG:40 | 155 |  |
| TBC1D1_Exon1 | rs2279027 | Ser14Pro | ref=T | TH_715069001_P_AG:26 | 100 |  |
| TBC1D1_Exon1 | rs2279027 | Ser14Pro | ref=T | TH_715086001_P_AG:50 | 152 |  |
| TBC1D1_Exon1 | rs2279027 | Ser14Pro | ref=T | TH_715089001_P_AG:47 | 130 |  |
| TBC1D1_Exon1 | rs2279027 | Ser14Pro | ref=T | TH_715092001_P_AG:41 | 194 |  |
| TBC1D1_Exon1 | rs2279027 | Ser14Pro | ref=T |  | 111 |  |
| TBC1D1_Exon1 | rs2279027 | Ser14Pro | ref=T | TH_715106001_P_AG:50 | 198 |  |
| TBC1D1_Exon1 | rs2279027 | Ser14Pro | ref=T | TH_715184001_P_AG:40 | 273 |  |
| TBC1D1_Exon1 | rs2279027 | Ser14Pro | ref=T | TH_4018001001_C_AG:42 | 156 |  |
| TBC1D1_Exon1 | rs2279027 | Ser14Pro | ref=T |  | 105 |  |
| TBC1D1_Exon1 | rs2279027 | Ser14Pro | ref=T | TH_4018004001_C_AG:42 | 184 |  |
| TBC1D1_Exon1 | rs2279027 | Ser14Pro | ref=T |  | 117 |  |
| TBC1D1_Exon1 | rs2279027 | Ser14Pro | ref=T |  | 114 |  |
| TBC1D1_Exon1 | rs2279027 | Ser14Pro | ref=T | TH_4018010001_C_AG:42 | 287 |  |
| TBC1D1_Exon1 | rs2279027 | Ser14Pro | ref=T |  | 102 |  |
| TBC1D1_Exon1 | rs2279027 | Ser14Pro | ref=T |  | 129 |  |
| TBC1D1_Exon1 | rs2279027 | Ser14Pro | ref=T |  | 250 |  |
| TBC1D1_Exon1 | rs2279027 | Ser14Pro | ref=T | TH_4018027001_C_AG:48 | 336 |  |
| TBC1D1_Exon1 | rs2279027 | Ser14Pro | ref=T | TH_4018046001_C_AG:48 | 328 |  |
| TBC1D1_Exon1 | rs2279027 | Ser14Pro | ref=T | TH_4018050001_C_AG:37 | 106 |  |
| TBC1D1_Exon1 | rs2279027 | Ser14Pro | ref=T |  | 132 |  |
| TBC1D1_Exon1 | rs2279027 | Ser14Pro | ref=T |  | 117 |  |
| TBC1D1_Exon1 | rs2279027 | Ser14Pro | ref=T | TH_4018054001_C_AG:41 | 152 |  |
| TBC1D1_Exon1 | rs2279027 | Ser14Pro | ref=T | TH_4018057001_C_AG:50 | 172 |  |
| TBC1D1_Exon1 | rs2279027 | Ser14Pro | ref=T | TH_4018058001_C_AG:38 | 127 |  |
| TBC1D1_Exon1 | rs2279027 | Ser14Pro | ref=T | TH_4018066001_C_AG:43 | 304 |  |
| TBC1D1_Exon1 | rs2279027 | Ser14Pro | ref=T | TH_418117001_C_AG:41 | 229 |  |
| TBC1D1_Exon1 | rs2279027 | Ser14Pro | ref=T |  | 138 |  |
| TBC1D1_Exon1 | rs2279027 | Ser14Pro | ref=T |  | 126 |  |
| TBC1D1_Exon1 | rs2279027 | Ser14Pro | ref=T | TH_418123001_C_AG:29 | 188 |  |
| TBC1D1_Exon1 | rs2279027 | Ser14Pro | ref=T | TH_418124001_C_AG:43 | 174 |  |
| TBC1D1_Exon1 | rs2279027 | Ser14Pro | ref=T | TH_418127001_C_AG:42 | 273 |  |
| TBC1D1_Exon1 | rs2279027 | Ser14Pro | ref=T | TH_418128001_C_AG:41 | 249 |  |
| TBC1D1_Exon1 | rs2279027 | Ser14Pro | ref=T | TH_418132001_C_AG:45 | 105 |  |
| TBC1D1_Exon1 | rs2279027 | Ser14Pro | ref=T | TH_418135001_C_AG:36 | 175 |  |
| TBC1D1_Exon1 | rs2279027 | Ser14Pro | ref=T |  | 111 |  |
| TBC1D1_Exon1 | rs2279027 | Ser14Pro | ref=T | TH_418137001_C_AG:46 | 194 |  |
| TBC1D1_Exon1 | rs2279027 | Ser14Pro | ref=T | TH_418139001_C_AG:38 | 161 |  |
| TBC1D1_Exon1 | rs2279027 | Ser14Pro | ref=T | TH_418141001_C_AG:36 | 119 |  |
| TBC1D1_Exon1 | rs2279027 | Ser14Pro | ref=T |  | 126 |  |
| TBC1D1_Exon1 | rs2279027 | Ser14Pro | ref=T | TH_418144001_C_AG:36 | 168 |  |
| TBC1D1_Exon1 | rs2279027 | Ser14Pro | ref=T | TH_418146001_C_AG:45 | 217 |  |
| TBC1D1_Exon1 | rs2279027 | Ser14Pro | ref=T | TH_418148001_C_AG:24 | 135 |  |
| TBC1D1_Exon1 | rs2279027 | Ser14Pro | ref=T | TH_418156001_C_AG:32 | 164 |  |
| TBC1D1_Exon1 | rs2279027 | Ser14Pro | ref=T | TH_418159001_C_AG:33 | 119 |  |
| TBC1D1_Exon1 | rs2279027 | Ser14Pro | ref=T | TH_418163001_C_AG:46 | 119 |  |
| TBC1D1_Exon1 | rs2279027 | Ser14Pro | ref=T |  | 117 |  |
| TBC1D1_Exon1 | rs2279027 | Ser14Pro | ref=T | TH_418165001_C_AG:50 | 244 |  |
| TBC1D1_Exon1 | rs2279027 | Ser14Pro | ref=T |  | 132 |  |
| TBC1D1_Exon1 | rs2279027 | Ser14Pro | ref=T |  | 132 |  |
| TBC1D1_Exon1 | rs2279027 | Ser14Pro | ref=T | TH_418171001_C_AG:35 | 253 |  |
| TBC1D1_Exon1 | rs2279027 | Ser14Pro | ref=T | TH_418178001_C_AG:34 | 145 |  |
| TBC1D1_Exon1 | rs2279027 | Ser14Pro | ref=T | TH_418182001_C_AG:47 | 162 |  |
| TBC1D1_Exon1 | rs2279027 | Ser14Pro | ref=T | TH_418195001_C_AG:35 | 196 |  |
| TBC1D1_Exon1 | rs2279027 | Ser14Pro | ref=T | TH_418198001_C_AG:44 | 146 |  |
| TBC1D1_Exon1 | rs2279027 | Ser14Pro | ref=T | TH_418300001_C_AG:46 | 369 |  |
| TBC1D1_Exon1 | rs2279027 | Ser14Pro | ref=T | TH_418304001_C_AG:47 | 338 |  |
| TBC1D1_Exon1 | rs2279027 | Ser14Pro | ref=T | TH_418344001_C_AG:50 | 180 |  |
| TBC1D1_Exon1 | rs2279027 | Ser14Pro | ref=T |  | 114 |  |
| TBC1D1_Exon1 | rs2279027 | Ser14Pro | ref=T | TH_418357001_C_AG:34 | 193 |  |
| TBC1D1_Exon1 | rs2279027 | Ser14Pro | ref=T | TH_418358001_C_AG:47 | 193 |  |
| TBC1D1_Exon1 | rs2279027 | Ser14Pro | ref=T | TH_418360001_C_AG:41 | 232 |  |
| TBC1D1_Exon1 | rs2279027 | Ser14Pro | ref=T | TH_418361001_C_AG:36 | 138 |  |
| TBC1D1_Exon1 | rs2279027 | Ser14Pro | ref=T |  | 193 |  |
| TBC1D1_Exon1 | rs2279027 | Ser14Pro | ref=T | TH_418692001_C_AG:48 | 260 |  |
| TBC1D1_Exon1 | rs2279027 | Ser14Pro | ref=T |  | 105 |  |
| TBC1D1_Exon1 | rs2279027 | Ser14Pro | ref=T |  | 135 |  |
| TBC1D1_Exon1 | rs2279027 | Ser14Pro | ref=T | TH_418748001_C_AG:44 | 351 |  |
| TBC1D1_Exon1 | rs145529203 | Asp121= | ref=C | hetero=TH_715104001_P_AG:41 | score=188 |  |
| TBC1D1_Exon1 | rs2279026 | Ala122= | ref=T | hetero=TH_1315006001_P_AG:50 | score=104 |  |
| TBC1D1_Exon1 | rs2279026 | Ala122= | ref=T | TH_1315318001_P_AG:44 | 158 |  |
| TBC1D1_Exon1 | rs2279026 | Ala122= | ref=T | TH_1415010001_P_AG:31 | 105 |  |
| TBC1D1_Exon1 | rs2279026 | Ala122= | ref=T | TH_1415115001_P_AG:34 | 172 |  |
| TBC1D1_Exon1 | rs2279026 | Ala122= | ref=T | TH_1415117001_P_AG:38 | 238 |  |
| TBC1D1_Exon1 | rs2279026 | Ala122= | ref=T | TH_1415121001_P_AG:48 | 305 |  |
| TBC1D1_Exon1 | rs2279026 | Ala122= | ref=T | TH_1415146001_P_AG:47 | 171 |  |
| TBC1D1_Exon1 | rs2279026 | Ala122= | ref=T | TH_1415154001_P_AG:46 | 199 |  |
| TBC1D1_Exon1 | rs2279026 | Ala122= | ref=T | TH_1715003001_P_AG:49 | 303 |  |
| TBC1D1_Exon1 | rs2279026 | Ala122= | ref=T | TH_1715005001_P_AG:43 | 217 |  |
| TBC1D1_Exon1 | rs2279026 | Ala122= | ref=T | TH_1715012001_P_AG:43 | 256 |  |
| TBC1D1_Exon1 | rs2279026 | Ala122= | ref=T |  | 126 |  |
| TBC1D1_Exon1 | rs2279026 | Ala122= | ref=T | TH_1715022001_P_AG:39 | 269 |  |
| TBC1D1_Exon1 | rs2279026 | Ala122= | ref=T | TH_1715029001_P_AG:40 | 129 |  |
| TBC1D1_Exon1 | rs2279026 | Ala122= | ref=T |  | 105 |  |
| TBC1D1_Exon1 | rs2279026 | Ala122= | ref=T | TH_1715034001_P_AG:41 | 310 |  |
| TBC1D1_Exon1 | rs2279026 | Ala122= | ref=T | TH_1715050001_P_AG:50 | 287 |  |
| TBC1D1_Exon1 | rs2279026 | Ala122= | ref=T | TH_1715051001_P_AG:35 | 191 |  |
| TBC1D1_Exon1 | rs2279026 | Ala122= | ref=T | TH_1715053001_P_AG:32 | 206 |  |
| TBC1D1_Exon1 | rs2279026 | Ala122= | ref=T | TH_1715067001_P_AG:50 | 221 |  |
| TBC1D1_Exon1 | rs2279026 | Ala122= | ref=T |  | 108 |  |
| TBC1D1_Exon1 | rs2279026 | Ala122= | ref=T | TH_1715091001_P_AG:42 | 154 |  |
| TBC1D1_Exon1 | rs2279026 | Ala122= | ref=T | TH_1815247001_P_AG:50 | 317 |  |
| TBC1D1_Exon1 | rs2279026 | Ala122= | ref=T | TH_1815251001_P_AG:38 | 226 |  |
| TBC1D1_Exon1 | rs2279026 | Ala122= | ref=T | TH_1815261001_P_AG:46 | 255 |  |
| TBC1D1_Exon1 | rs2279026 | Ala122= | ref=T | TH_1815313001_P_AG:50 | 186 |  |
| TBC1D1_Exon1 | rs2279026 | Ala122= | ref=T | TH_1815408001_P_AG:45 | 253 |  |
| TBC1D1_Exon1 | rs2279026 | Ala122= | ref=T | TH_1815484001_P_AG:49 | 327 |  |
| TBC1D1_Exon1 | rs2279026 | Ala122= | ref=T | TH_515015001_P_AG:47 | 294 |  |
| TBC1D1_Exon1 | rs2279026 | Ala122= | ref=T | TH_515016001_P_AG:42 | 122 |  |
| TBC1D1_Exon1 | rs2279026 | Ala122= | ref=T | TH_515018001_P_AG:33 | 162 |  |
| TBC1D1_Exon1 | rs2279026 | Ala122= | ref=T | TH_515055001_P_AG:50 | 172 |  |
| TBC1D1_Exon1 | rs2279026 | Ala122= | ref=T | TH_515062001_P_AG:36 | 179 |  |
| TBC1D1_Exon1 | rs2279026 | Ala122= | ref=T | TH_515074001_P_AG:48 | 234 |  |
| TBC1D1_Exon1 | rs2279026 | Ala122= | ref=T |  | 238 |  |
| TBC1D1_Exon1 | rs2279026 | Ala122= | ref=T | TH_515114001_P_AG:42 | 287 |  |
| TBC1D1_Exon1 | rs2279026 | Ala122= | ref=T | TH_515121001_P_AG:48 | 287 |  |
| TBC1D1_Exon1 | rs2279026 | Ala122= | ref=T |  | 102 |  |
| TBC1D1_Exon1 | rs2279026 | Ala122= | ref=T | TH_515162001_P_AG:45 | 327 |  |
| TBC1D1_Exon1 | rs2279026 | Ala122= | ref=T | TH_515175001_P_AG:37 | 162 |  |
| TBC1D1_Exon1 | rs2279026 | Ala122= | ref=T | TH_515185001_P_AG:48 | 223 |  |
| TBC1D1_Exon1 | rs2279026 | Ala122= | ref=T | TH_515201001_P_AG:38 | 176 |  |
| TBC1D1_Exon1 | rs2279026 | Ala122= | ref=T | TH_515244001_P_AG:39 | 245 |  |
| TBC1D1_Exon1 | rs2279026 | Ala122= | ref=T |  | 105 |  |
| TBC1D1_Exon1 | rs2279026 | Ala122= | ref=T | TH_515294001_P_AG:46 | 334 |  |
| TBC1D1_Exon1 | rs2279026 | Ala122= | ref=T | TH_515333001_P_AG:44 | 206 |  |
| TBC1D1_Exon1 | rs2279026 | Ala122= | ref=T | TH_715063001_P_AG:40 | 120 |  |
| TBC1D1_Exon1 | rs2279026 | Ala122= | ref=T | TH_715069001_P_AG:26 | 101 |  |
| TBC1D1_Exon1 | rs2279026 | Ala122= | ref=T | TH_715088001_P_AG:45 | 202 |  |
| TBC1D1_Exon1 | rs2279026 | Ala122= | ref=T | TH_715104001_P_AG:41 | 187 |  |
| TBC1D1_Exon1 | rs2279026 | Ala122= | ref=T | TH_715184001_P_AG:47 | 300 |  |
| TBC1D1_Exon1 | rs2279026 | Ala122= | ref=T | TH_4018004001_C_AG:37 | 161 |  |
| TBC1D1_Exon1 | rs2279026 | Ala122= | ref=T | TH_4018009001_C_AG:32 | 187 |  |
| TBC1D1_Exon1 | rs2279026 | Ala122= | ref=T | TH_4018010001_C_AG:41 | 300 |  |
| TBC1D1_Exon1 | rs2279026 | Ala122= | ref=T | TH_4018012001_C_AG:45 | 232 |  |
| TBC1D1_Exon1 | rs2279026 | Ala122= | ref=T | TH_4018015001_C_AG:33 | 159 |  |
| TBC1D1_Exon1 | rs2279026 | Ala122= | ref=T | TH_4018024001_C_AG:50 | 327 |  |
| TBC1D1_Exon1 | rs2279026 | Ala122= | ref=T | TH_4018052001_C_AG:43 | 229 |  |
| TBC1D1_Exon1 | rs2279026 | Ala122= | ref=T | TH_418117001_C_AG:45 | 253 |  |
| TBC1D1_Exon1 | rs2279026 | Ala122= | ref=T | TH_418121001_C_AG:29 | 188 |  |
| TBC1D1_Exon1 | rs2279026 | Ala122= | ref=T | TH_418122001_C_AG:45 | 263 |  |
| TBC1D1_Exon1 | rs2279026 | Ala122= | ref=T | TH_418124001_C_AG:41 | 217 |  |
| TBC1D1_Exon1 | rs2279026 | Ala122= | ref=T | TH_418127001_C_AG:41 | 275 |  |
| TBC1D1_Exon1 | rs2279026 | Ala122= | ref=T | TH_418133001_C_AG:46 | 201 |  |
| TBC1D1_Exon1 | rs2279026 | Ala122= | ref=T | TH_418134001_C_AG:35 | 108 |  |
| TBC1D1_Exon1 | rs2279026 | Ala122= | ref=T | TH_418135001_C_AG:43 | 209 |  |
| TBC1D1_Exon1 | rs2279026 | Ala122= | ref=T | TH_418136001_C_AG:46 | 231 |  |
| TBC1D1_Exon1 | rs2279026 | Ala122= | ref=T | TH_418143001_C_AG:33 | 139 |  |
| TBC1D1_Exon1 | rs2279026 | Ala122= | ref=T | TH_418144001_C_AG:33 | 122 |  |
| TBC1D1_Exon1 | rs2279026 | Ala122= | ref=T | TH_418152001_C_AG:50 | 118 |  |
| TBC1D1_Exon1 | rs2279026 | Ala122= | ref=T | TH_418157001_C_AG:47 | 162 |  |
| TBC1D1_Exon1 | rs2279026 | Ala122= | ref=T | TH_418164001_C_AG:37 | 229 |  |
| TBC1D1_Exon1 | rs2279026 | Ala122= | ref=T | TH_418170001_C_AG:48 | 303 |  |
| TBC1D1_Exon1 | rs2279026 | Ala122= | ref=T | TH_418171001_C_AG:21 | 126 |  |
| TBC1D1_Exon1 | rs2279026 | Ala122= | ref=T | TH_418175001_C_AG:36 | 153 |  |
| TBC1D1_Exon1 | rs2279026 | Ala122= | ref=T | TH_418185001_C_AG:40 | 166 |  |
| TBC1D1_Exon1 | rs2279026 | Ala122= | ref=T | TH_418196001_C_AG:45 | 167 |  |
| TBC1D1_Exon1 | rs2279026 | Ala122= | ref=T | TH_418304001_C_AG:35 | 280 |  |
| TBC1D1_Exon1 | rs2279026 | Ala122= | ref=T | TH_418359001_C_AG:36 | 134 |  |
| TBC1D1_Exon1 | rs2279026 | Ala122= | ref=T | TH_418683001_C_AG:47 | 289 |  |
| TBC1D1_Exon1 | rs2279026 | Ala122= | ref=T |  | 120 |  |
| TBC1D1_Exon1 | rs35859249 | Arg125Trp | ref=C | TH_1715012001_P_AG:40 | 237 |  |
| TBC1D1_Exon1 | rs35859249 | Arg125Trp | ref=C | TH_1715015001_P_AG:46 | 224 |  |
| TBC1D1_Exon1 | rs35859249 | Arg125Trp | ref=C | TH_1715030001_P_AG:48 | 205 |  |
| TBC1D1_Exon1 | rs35859249 | Arg125Trp | ref=C | TH_1715034001_P_AG:40 | 306 |  |
| TBC1D1_Exon1 | rs35859249 | Arg125Trp | ref=C | TH_1715051001_P_AG:37 | 211 |  |
| TBC1D1_Exon1 | rs35859249 | Arg125Trp | ref=C | TH_1715067001_P_AG:41 | 202 |  |
| TBC1D1_Exon1 | rs35859249 | Arg125Trp | ref=C | TH_1715093001_P_AG:29 | 119 |  |
| TBC1D1_Exon1 | rs35859249 | Arg125Trp | ref=C | TH_1815313001_P_AG:50 | 191 |  |
| TBC1D1_Exon1 | rs35859249 | Arg125Trp | ref=C | TH_1815408001_P_AG:41 | 257 |  |
| TBC1D1_Exon1 | rs35859249 | Arg125Trp | ref=C | TH_1815410001_P_AG:43 | 144 |  |
| TBC1D1_Exon1 | rs35859249 | Arg125Trp | ref=C | TH_1815484001_P_AG:45 | 305 |  |
| TBC1D1_Exon1 | rs35859249 | Arg125Trp | ref=C | TH_515016001_P_AG:40 | 118 |  |
| TBC1D1_Exon1 | rs35859249 | Arg125Trp | ref=C | TH_515055001_P_AG:45 | 156 |  |
| TBC1D1_Exon1 | rs35859249 | Arg125Trp | ref=C | TH_515074001_P_AG:45 | 229 |  |
| TBC1D1_Exon1 | rs35859249 | Arg125Trp | ref=C | TH_515114001_P_AG:42 | 282 |  |
| TBC1D1_Exon1 | rs35859249 | Arg125Trp | ref=C | TH_515121001_P_AG:49 | 302 |  |
| TBC1D1_Exon1 | rs35859249 | Arg125Trp | ref=C | TH_515132001_P_AG:34 | 130 |  |
| TBC1D1_Exon1 | rs35859249 | Arg125Trp | ref=C | TH_515162001_P_AG:50 | 341 |  |
| TBC1D1_Exon1 | rs35859249 | Arg125Trp | ref=C | TH_515185001_P_AG:44 | 204 |  |
| TBC1D1_Exon1 | rs35859249 | Arg125Trp | ref=C | TH_515333001_P_AG:44 | 197 |  |
| TBC1D1_Exon1 | rs35859249 | Arg125Trp | ref=C | TH_715088001_P_AG:47 | 202 |  |
| TBC1D1_Exon1 | rs35859249 | Arg125Trp | ref=C | TH_715184001_P_AG:48 | 296 |  |
| TBC1D1_Exon1 | rs35859249 | Arg125Trp | ref=C | TH_4018004001_C_AG:40 | 181 |  |
| TBC1D1_Exon1 | rs35859249 | Arg125Trp | ref=C | TH_4018012001_C_AG:47 | 232 |  |
| TBC1D1_Exon1 | rs35859249 | Arg125Trp | ref=C | TH_4018015001_C_AG:39 | 202 |  |
| TBC1D1_Exon1 | rs35859249 | Arg125Trp | ref=C |  | 102 |  |
| TBC1D1_Exon1 | rs35859249 | Arg125Trp | ref=C | TH_418117001_C_AG:39 | 216 |  |
| TBC1D1_Exon1 | rs35859249 | Arg125Trp | ref=C | TH_418122001_C_AG:41 | 243 |  |
| TBC1D1_Exon1 | rs35859249 | Arg125Trp | ref=C | TH_418127001_C_AG:41 | 269 |  |
| TBC1D1_Exon1 | rs35859249 | Arg125Trp | ref=C | TH_418134001_C_AG:33 | 106 |  |
| TBC1D1_Exon1 | rs35859249 | Arg125Trp | ref=C | TH_418136001_C_AG:44 | 218 |  |
| TBC1D1_Exon1 | rs35859249 | Arg125Trp | ref=C | TH_418143001_C_AG:36 | 164 |  |
| TBC1D1_Exon1 | rs35859249 | Arg125Trp | ref=C | TH_418152001_C_AG:50 | 119 |  |
| TBC1D1_Exon1 | rs35859249 | Arg125Trp | ref=C | TH_418157001_C_AG:50 | 165 |  |
| TBC1D1_Exon1 | rs35859249 | Arg125Trp | ref=C | TH_418164001_C_AG:43 | 254 |  |
| TBC1D1_Exon1 | rs35859249 | Arg125Trp | ref=C | TH_418171001_C_AG:22 | 145 |  |
| TBC1D1_Exon1 | rs35859249 | Arg125Trp | ref=C | TH_418175001_C_AG:32 | 144 |  |
| TBC1D1_Exon1 | rs35859249 | Arg125Trp | ref=C | TH_418196001_C_AG:47 | 198 |  |
| TBC1D1_Exon1 | rs35859249 | Arg125Trp | ref=C | TH_418304001_C_AG:38 | 298 |  |
| TBC1D1_Exon1 | rs35859249 | Arg125Trp | ref=C | TH_418683001_C_AG:47 | 288 |  |
| TBC1D1_Exon12 | rs145177739 | Gln619Arg | ref=A | hetero=TH_515039001_P_AG:38 | score=221 |  |
| TBC1D1_Exon14 | rs58983546 | Arg695Cys | ref=C | hetero=TH_1315304001_P_AG:48 | score=285 |  |
| TBC1D1_Exon14 | rs58983546 | Arg695Cys | ref=C | TH_1315376001_P_AG:33 | 136 |  |
| TBC1D1_Exon14 | rs58983546 | Arg695Cys | ref=C | TH_1415120001_P_AG:31 | 244 |  |
| TBC1D1_Exon14 | rs58983546 | Arg695Cys | ref=C | TH_1415146001_P_AG:40 | 182 |  |
| TBC1D1_Exon14 | rs58983546 | Arg695Cys | ref=C | TH_1715012001_P_AG:40 | 257 |  |
| TBC1D1_Exon14 | rs58983546 | Arg695Cys | ref=C | TH_1715065001_P_AG:50 | 249 |  |
| TBC1D1_Exon14 | rs58983546 | Arg695Cys | ref=C | TH_1715070001_P_AG:50 | 121 |  |
| TBC1D1_Exon14 | rs58983546 | Arg695Cys | ref=C | TH_1715087001_P_AG:48 | 231 |  |
| TBC1D1_Exon14 | rs58983546 | Arg695Cys | ref=C | TH_1715090001_P_AG:40 | 189 |  |
| TBC1D1_Exon14 | rs58983546 | Arg695Cys | ref=C | TH_1715091001_P_AG:46 | 224 |  |
| TBC1D1_Exon14 | rs58983546 | Arg695Cys | ref=C | TH_1715093001_P_AG:46 | 207 |  |
| TBC1D1_Exon14 | rs58983546 | Arg695Cys | ref=C | TH_1715101001_P_AG:46 | 212 |  |
| TBC1D1_Exon14 | rs58983546 | Arg695Cys | ref=C | TH_1815221001_P_AG:44 | 250 |  |
| TBC1D1_Exon14 | rs58983546 | Arg695Cys | ref=C | TH_1815231001_P_AG:32 | 131 |  |
| TBC1D1_Exon14 | rs58983546 | Arg695Cys | ref=C | TH_1815278001_P_AG:47 | 341 |  |
| TBC1D1_Exon14 | rs58983546 | Arg695Cys | ref=C | TH_1815313001_P_AG:41 | 235 |  |
| TBC1D1_Exon14 | rs58983546 | Arg695Cys | ref=C | TH_1815333001_P_AG:43 | 180 |  |
| TBC1D1_Exon14 | rs58983546 | Arg695Cys | ref=C | TH_1815349001_P_AG:45 | 300 |  |
| TBC1D1_Exon14 | rs58983546 | Arg695Cys | ref=C | TH_1815443001_P_AG:45 | 199 |  |
| TBC1D1_Exon14 | rs58983546 | Arg695Cys | ref=C | TH_515003001_P_AG:43 | 238 |  |
| TBC1D1_Exon14 | rs58983546 | Arg695Cys | ref=C | TH_515016001_P_AG:34 | 170 |  |
| TBC1D1_Exon14 | rs58983546 | Arg695Cys | ref=C | TH_515037001_P_AG:42 | 339 |  |
| TBC1D1_Exon14 | rs58983546 | Arg695Cys | ref=C | TH_515185001_P_AG:37 | 164 |  |
| TBC1D1_Exon14 | rs58983546 | Arg695Cys | ref=C | TH_515223001_P_AG:39 | 265 |  |
| TBC1D1_Exon14 | rs58983546 | Arg695Cys | ref=C | TH_515285001_P_AG:46 | 272 |  |
| TBC1D1_Exon14 | rs58983546 | Arg695Cys | ref=C | TH_515296001_P_AG:48 | 245 |  |
| TBC1D1_Exon14 | rs58983546 | Arg695Cys | ref=C | TH_715006001_P_AG:38 | 171 |  |
| TBC1D1_Exon14 | rs58983546 | Arg695Cys | ref=C | TH_715088001_P_AG:36 | 158 |  |
| TBC1D1_Exon14 | rs58983546 | Arg695Cys | ref=C | TH_4018003001_C_AG:50 | 303 |  |
| TBC1D1_Exon14 | rs58983546 | Arg695Cys | ref=C | TH_4018005001_C_AG:45 | 271 |  |
| TBC1D1_Exon14 | rs58983546 | Arg695Cys | ref=C |  | 156 |  |
| TBC1D1_Exon14 | rs58983546 | Arg695Cys | ref=C | TH_4018023001_C_AG:47 | 323 |  |
| TBC1D1_Exon14 | rs58983546 | Arg695Cys | ref=C | TH_4018046001_C_AG:45 | 335 |  |
| TBC1D1_Exon14 | rs58983546 | Arg695Cys | ref=C | TH_4018050001_C_AG:43 | 176 |  |
| TBC1D1_Exon14 | rs58983546 | Arg695Cys | ref=C | TH_4018051001_C_AG:45 | 307 |  |
| TBC1D1_Exon14 | rs58983546 | Arg695Cys | ref=C | TH_4018057001_C_AG:43 | 217 |  |
| TBC1D1_Exon14 | rs58983546 | Arg695Cys | ref=C | TH_4018095001_C_AG:45 | 158 |  |
| TBC1D1_Exon14 | rs58983546 | Arg695Cys | ref=C | TH_418121001_C_AG:50 | 258 |  |
| TBC1D1_Exon14 | rs58983546 | Arg695Cys | ref=C | TH_418125001_C_AG:38 | 281 |  |
| TBC1D1_Exon14 | rs58983546 | Arg695Cys | ref=C | TH_418134001_C_AG:48 | 241 |  |
| TBC1D1_Exon14 | rs58983546 | Arg695Cys | ref=C | TH_418139001_C_AG:38 | 187 |  |
| TBC1D1_Exon14 | rs58983546 | Arg695Cys | ref=C | TH_418141001_C_AG:50 | 147 |  |
| TBC1D1_Exon14 | rs58983546 | Arg695Cys | ref=C | TH_418144001_C_AG:44 | 289 |  |
| TBC1D1_Exon14 | rs58983546 | Arg695Cys | ref=C | TH_418146001_C_AG:44 | 215 |  |
| TBC1D1_Exon14 | rs58983546 | Arg695Cys | ref=C | TH_418147001_C_AG:39 | 199 |  |
| TBC1D1_Exon14 | rs58983546 | Arg695Cys | ref=C | TH_418170001_C_AG:42 | 277 |  |
| TBC1D1_Exon14 | rs58983546 | Arg695Cys | ref=C | TH_418175001_C_AG:38 | 124 |  |
| TBC1D1_Exon14 | rs58983546 | Arg695Cys | ref=C | TH_418300001_C_AG:32 | 214 |  |
| TBC1D1_Exon14 | rs58983546 | Arg695Cys | ref=C | TH_418302001_C_AG:39 | 196 |  |
| TBC1D1_Exon14 | rs58983546 | Arg695Cys | ref=C | TH_418361001_C_AG:43 | 236 |  |
| TBC1D1_Exon14 | rs58983546 | Arg695Cys | ref=C | TH_418683001_C_AG:49 | 309 |  |
| TBC1D1_Exon14 | rs58983546 | Arg695Cys | ref=C | TH_418739001_C_AG:37 | 268 |  |
| TBC1D1_Exon14 | rs58983546 | Arg695Cys | ref=C | TH_418748001_C_AG:45 | 366 |  |
| TBC1D1_Exon16 | rs16994165 | Ser159= | ref=G | TH_1715101001_P_AG:45 | 106 |  |
| TBC1D1_Exon16 | rs16994165 | Ser159= | ref=G | TH_1815221001_P_AG:33 | 132 |  |
| TBC1D1_Exon16 | rs16994165 | Ser159= | ref=G | TH_1815251001_P_AG:44 | 188 |  |
| TBC1D1_Exon16 | rs16994165 | Ser159= | ref=G | TH_1815261001_P_AG:39 | 158 |  |
| TBC1D1_Exon16 | rs16994165 | Ser159= | ref=G | TH_515091001_P_AG:37 | 106 |  |
| TBC1D1_Exon16 | rs16994165 | Ser159= | ref=G | TH_515095001_P_AG:44 | 134 |  |
| TBC1D1_Exon16 | rs16994165 | Ser159= | ref=G | TH_515121001_P_AG:47 | 201 |  |
| TBC1D1_Exon16 | rs16994165 | Ser159= | ref=G | TH_515175001_P_AG:50 | 123 |  |
| TBC1D1_Exon16 | rs16994165 | Ser159= | ref=G | TH_515178001_P_AG:50 | 143 |  |
| TBC1D1_Exon16 | rs16994165 | Ser159= | ref=G | TH_515262001_P_AG:33 | 122 |  |
| TBC1D1_Exon16 | rs16994165 | Ser159= | ref=G |  | 108 |  |
| TBC1D1_Exon16 | rs16994165 | Ser159= | ref=G | TH_715071001_P_AG:50 | 125 |  |
| TBC1D1_Exon16 | rs16994165 | Ser159= | ref=G | TH_715184001_P_AG:37 | 183 |  |
| TBC1D1_Exon16 | rs16994165 | Ser159= | ref=G | TH_4018012001_C_AG:42 | 203 |  |
| TBC1D1_Exon16 | rs16994165 | Ser159= | ref=G | TH_4018046001_C_AG:48 | 296 |  |
| TBC1D1_Exon16 | rs16994165 | Ser159= | ref=G | TH_418122001_C_AG:41 | 132 |  |
| TBC1D1_Exon16 | rs16994165 | Ser159= | ref=G | TH_418133001_C_AG:43 | 143 |  |
| TBC1D1_Exon16 | rs16994165 | Ser159= | ref=G | TH_418167001_C_AG:38 | 113 |  |
| TBC1D1_Exon19 |  | Leu838Val | ref=T | hetero=TH_515035001_P_AG:48 | score=253 |  |
| TBC1D1_Exon23 |  | Arg1091His | ref=G | hetero=TH_1715040001_P_AG:47 | score=182 |  |
| TBC1D1_Exon24 | rs13110318 | Arg1136Gln | ref=G | hetero=TH_1715034001_P_AG:42 | score=175 |  |
| TBC1D1_Exon24 | rs13110318 | Arg1136Gln | ref=G | TH_515035001_P_AG:40 | 116 |  |
| TBC1D1_Exon24 | rs13110318 | Arg1136Gln | ref=G | TH_515095001_P_AG:42 | 106 |  |
| TBC1D1_Exon24 | rs13110318 | Arg1136Gln | ref=G | TH_515215001_P_AG:46 | 134 |  |
| TBC1D1_Exon24 | rs13110318 | Arg1136Gln | ref=G | TH_515221001_P_AG:40 | 104 |  |
| TBC1D1_Exon24 | rs13110318 | Arg1136Gln | ref=G | TH_418136001_C_AG:42 | 157 |  |
| TBC1D1_Exon6 | rs112261209 | Arg327Lys | ref=G | hetero=TH_1715034001_P_AG:50 | score=365 |  |
| TBC1D1_Exon6 | rs112261209 | Arg327Lys | ref=G | TH_515099001_P_AG:47 | 178 |  |
| TBC1D1_Exon6 | rs112261209 | Arg327Lys | ref=G | TH_515148001_P_AG:50 | 140 |  |
| TBC1D1_Exon6 | rs112261209 | Arg327Lys | ref=G | TH_715006001_P_AG:31 | 100 |  |
| TBC1D1_Exon6 | rs112261209 | Arg327Lys | ref=G | TH_715039001_P_AG:40 | 117 |  |
| TBC1D1_Exon6 | rs112261209 | Arg327Lys | ref=G | TH_4018015001_C_AG:50 | 167 |  |
| TBC1D1_Exon6 | rs112261209 | Arg327Lys | ref=G | TH_418157001_C_AG:47 | 157 |  |
| TBC1D1_Exon6 | rs112261209 | Arg327Lys | ref=G | TH_418360001_C_AG:50 | 319 |  |
| TBC1D1_Exon7 | rs61731607 | Ala384Pro | ref=G | hetero=TH_1715003001_P_AG:40 | score=171 |  |
| TBC1D1_Exon7 | rs61731607 | Ala384Pro | ref=G | TH_1715051001_P_AG:43 | 128 |  |
| TBC1D1_Exon7 | rs61731607 | Ala384Pro | ref=G | TH_1815099001_P_AG:31 | 110 |  |
| TBC1D1_Exon7 | rs61731607 | Ala384Pro | ref=G | TH_1815337001_P_AG:46 | 190 |  |
| TBC1D1_Exon7 | rs61731607 | Ala384Pro | ref=G | TH_515091001_P_AG:34 | 174 |  |
| TBC1D1_Exon7 | rs61731607 | Ala384Pro | ref=G | TH_715064001_P_AG:40 | 152 |  |
| TBC1D1_Exon7 | rs61731607 | Ala384Pro | ref=G | TH_715095001_P_AG:46 | 118 |  |
| TBC1D1_Exon7 | rs61731607 | Ala384Pro | ref=G | TH_418165001_C_AG:31 | 119 |  |
| TBC1D1_Exon7 | rs61731607 | Ala384Pro | ref=G | TH_418300001_C_AG:47 | 326 |  |
| TBC1D1_Exon7 | rs61731610 | Gly389Ser | ref=G | hetero=TH_1715003001_P_AG:40 | score=160 |  |
| TBC1D1_Exon7 | rs61731610 | Gly389Ser | ref=G | TH_1715051001_P_AG:50 | 142 |  |
| TBC1D1_Exon7 | rs61731610 | Gly389Ser | ref=G | TH_1815099001_P_AG:47 | 151 |  |
| TBC1D1_Exon7 | rs61731610 | Gly389Ser | ref=G | TH_1815337001_P_AG:43 | 170 |  |
| TBC1D1_Exon7 | rs61731610 | Gly389Ser | ref=G | TH_515091001_P_AG:41 | 200 |  |
| TBC1D1_Exon7 | rs61731610 | Gly389Ser | ref=G | TH_715064001_P_AG:34 | 103 |  |
| TBC1D1_Exon7 | rs61731610 | Gly389Ser | ref=G | TH_418165001_C_AG:33 | 105 |  |
| TBC1D1_Exon7 | rs61731610 | Gly389Ser | ref=G | TH_418300001_C_AG:41 | 305 |  |
| TBC1D1_Exon7 | rs34119528 | Pro391= | ref=C | TH_1415121001_P_AG:42 | 206 |  |
| TBC1D1_Exon7 | rs34119528 | Pro391= | ref=C | TH_1715012001_P_AG:50 | 157 |  |
| TBC1D1_Exon7 | rs34119528 | Pro391= | ref=C | TH_1715034001_P_AG:44 | 226 |  |
| TBC1D1_Exon7 | rs34119528 | Pro391= | ref=C | TH_1715090001_P_AG:47 | 160 |  |
| TBC1D1_Exon7 | rs34119528 | Pro391= | ref=C |  | 105 |  |
| TBC1D1_Exon7 | rs34119528 | Pro391= | ref=C | TH_515003001_P_AG:43 | 136 |  |
| TBC1D1_Exon7 | rs34119528 | Pro391= | ref=C | TH_515046001_P_AG:42 | 106 |  |
| TBC1D1_Exon7 | rs34119528 | Pro391= | ref=C | TH_515055001_P_AG:42 | 109 |  |
| TBC1D1_Exon7 | rs34119528 | Pro391= | ref=C | TH_515078001_P_AG:47 | 226 |  |
| TBC1D1_Exon7 | rs34119528 | Pro391= | ref=C | TH_515112001_P_AG:42 | 239 |  |
| TBC1D1_Exon7 | rs34119528 | Pro391= | ref=C | TH_515114001_P_AG:42 | 186 |  |
| TBC1D1_Exon7 | rs34119528 | Pro391= | ref=C |  | 150 |  |
| TBC1D1_Exon7 | rs34119528 | Pro391= | ref=C | TH_515262001_P_AG:47 | 155 |  |
| TBC1D1_Exon7 | rs34119528 | Pro391= | ref=C | TH_715184001_P_AG:41 | 199 |  |
| TBC1D1_Exon7 | rs34119528 | Pro391= | ref=C | TH_4018012001_C_AG:32 | 117 |  |
| TBC1D1_Exon7 | rs34119528 | Pro391= | ref=C | TH_4018015001_C_AG:46 | 114 |  |
| TBC1D1_Exon7 | rs34119528 | Pro391= | ref=C | TH_418119001_C_AG:50 | 158 |  |
| TBC1D1_Exon7 | rs34119528 | Pro391= | ref=C | TH_418127001_C_AG:40 | 157 |  |
| TBC1D1_Exon7 | rs34119528 | Pro391= | ref=C | TH_418137001_C_AG:38 | 100 |  |
| TBC1D1_Exon7 | rs34119528 | Pro391= | ref=C | TH_418143001_C_AG:46 | 113 |  |
| TBC1D1_Exon7 | rs34119528 | Pro391= | ref=C | TH_418147001_C_AG:42 | 149 |  |
| TBC1D1_Exon7 | rs34119528 | Pro391= | ref=C | TH_418164001_C_AG:43 | 183 |  |
| TBC1D1_Exon7 | rs34119528 | Pro391= | ref=C | TH_418178001_C_AG:46 | 130 |  |
| TBC1D1_Exon7 | rs34119528 | Pro391= | ref=C | TH_418344001_C_AG:46 | 104 |  |
| TBC1D1_Exon7 | rs34119528 | Pro391= | ref=C | TH_418361001_C_AG:36 | 116 |  |
| TBC1D1_Exon7 | rs34119528 | Pro391= | ref=C | TH_418701001_C_AG:44 | 215 |  |
| TBC1D1_Exon8 | rs112435875 | Leu418= | ref=G | hetero=TH_1715029001_P_AG:43 | score=138 |  |
| TBC1D1_Exon8 | rs112435875 | Leu418= | ref=G | TH_1715051001_P_AG:40 | 257 |  |
| TBC1D1_Exon8 | rs112435875 | Leu418= | ref=G | TH_1715053001_P_AG:47 | 301 |  |
| TBC1D1_Exon8 | rs112435875 | Leu418= | ref=G | TH_515062001_P_AG:41 | 145 |  |
| TBC1D1_Exon8 | rs112435875 | Leu418= | ref=G | TH_515112001_P_AG:47 | 313 |  |
| TBC1D1_Exon8 | rs112435875 | Leu418= | ref=G | TH_515333001_P_AG:39 | 164 |  |
| TBC1D1_Exon8 | rs112435875 | Leu418= | ref=G | TH_715088001_P_AG:47 | 159 |  |
| TBC1D1_Exon8 | rs112435875 | Leu418= | ref=G | TH_715089001_P_AG:37 | 122 |  |
| TBC1D1_Exon8 | rs112435875 | Leu418= | ref=G | TH_4018097001_C_AG:40 | 103 |  |
| TBC1D1_Exon8 | rs112435875 | Leu418= | ref=G | TH_418166001_C_AG:25 | 104 |  |
| TBC1D1_Exon8 | rs112435875 | Leu418= | ref=G | TH_418171001_C_AG:23 | 169 |  |
| TBC1D1_Exon8 | rs112435875 | Leu418= | ref=G | TH_418178001_C_AG:42 | 200 |  |
| TBC1D1_Exon8 | rs112435875 | Leu418= | ref=G | TH_418304001_C_AG:41 | 291 |  |
| TBC1D1_Exon8 | rs112435875 | Leu418= | ref=G | TH_418344001_C_AG:47 | 211 |  |
| TBC1D1_Exon8 | rs112435875 | Leu418= | ref=G | TH_418683001_C_AG:48 | 273 |  |
| TBC1D1_Exon9 |  | Arg443Stop bzw. Sec | ref=C | hetero=TH_515078001_P_AG:37 | score=230 |  |
| TBC1D1_Exon9 |  | Glu468= | ref=G | hetero=TH_1315200001_P_AG:37 | score=172 |  |
| TMEM18_Exon1 | rs3210390 | Val17= | ref=C | TH_1815408001_P_AG:46 | 100 |  |
| TMEM18_Exon1 | rs3210390 | Val17= | ref=C | TH_418300001_C_AG:31 | 101 |  |
| TNKS_Exon1 | rs33985989 | Ser142= | ref=T | TH_1715034001_P_AG:43 | 203 |  |
| TNKS_Exon1 | rs33985989 | Ser142= | ref=T | TH_1815349001_P_AG:36 | 122 |  |
| TNKS_Exon1 | rs33985989 | Ser142= | ref=T | TH_418304001_C_AG:47 | 243 |  |
| TNKS_Exon1 | rs35433754 | Ser148= | ref=G | hetero=TH_1715022001_P_AG:38 | score=116 |  |
| TNKS_Exon1 | rs33945943 | Arg200= | ref=G | hetero=TH_1315304001_P_AG:36 | score=119 |  |
| TNKS_Exon1 | rs33945943 | Arg200= | ref=G | TH_1415120001_P_AG:43 | 113 |  |
| TNKS_Exon1 | rs33945943 | Arg200= | ref=G | TH_1715050001_P_AG:48 | 195 |  |
| TNKS_Exon1 | rs33945943 | Arg200= | ref=G | TH_1715105001_P_AG:47 | 125 |  |
| TNKS_Exon1 | rs33945943 | Arg200= | ref=G | TH_1815079001_P_AG:42 | 127 |  |
| TNKS_Exon1 | rs33945943 | Arg200= | ref=G | TH_1815131001_P_AG:44 | 140 |  |
| TNKS_Exon1 | rs33945943 | Arg200= | ref=G | TH_1815221001_P_AG:50 | 111 |  |
| TNKS_Exon1 | rs33945943 | Arg200= | ref=G | TH_1815261001_P_AG:47 | 124 |  |
| TNKS_Exon1 | rs33945943 | Arg200= | ref=G | TH_1815268001_P_AG:37 | 160 |  |
| TNKS_Exon1 | rs33945943 | Arg200= | ref=G | TH_1815284001_P_AG:39 | 131 |  |
| TNKS_Exon1 | rs33945943 | Arg200= | ref=G | TH_1815293001_P_AG:28 | 101 |  |
| TNKS_Exon1 | rs33945943 | Arg200= | ref=G | TH_1815337001_P_AG:43 | 173 |  |
| TNKS_Exon1 | rs33945943 | Arg200= | ref=G | TH_1815484001_P_AG:38 | 181 |  |
| TNKS_Exon1 | rs33945943 | Arg200= | ref=G | TH_515003001_P_AG:42 | 113 |  |
| TNKS_Exon1 | rs33945943 | Arg200= | ref=G | TH_515015001_P_AG:39 | 121 |  |
| TNKS_Exon1 | rs33945943 | Arg200= | ref=G | TH_515078001_P_AG:48 | 200 |  |
| TNKS_Exon1 | rs33945943 | Arg200= | ref=G | TH_515091001_P_AG:41 | 131 |  |
| TNKS_Exon1 | rs33945943 | Arg200= | ref=G |  | 111 |  |
| TNKS_Exon1 | rs33945943 | Arg200= | ref=G | TH_515151001_P_AG:50 | 175 |  |
| TNKS_Exon1 | rs33945943 | Arg200= | ref=G | TH_515162001_P_AG:41 | 221 |  |
| TNKS_Exon1 | rs33945943 | Arg200= | ref=G | TH_515164001_P_AG:33 | 142 |  |
| TNKS_Exon1 | rs33945943 | Arg200= | ref=G | TH_515165001_P_AG:43 | 267 |  |
| TNKS_Exon1 | rs33945943 | Arg200= | ref=G | TH_515206001_P_AG:47 | 147 |  |
| TNKS_Exon1 | rs33945943 | Arg200= | ref=G | TH_515213001_P_AG:38 | 119 |  |
| TNKS_Exon1 | rs33945943 | Arg200= | ref=G | TH_515247001_P_AG:50 | 286 |  |
| TNKS_Exon1 | rs33945943 | Arg200= | ref=G | TH_715006001_P_AG:40 | 109 |  |
| TNKS_Exon1 | rs33945943 | Arg200= | ref=G | TH_715184001_P_AG:48 | 222 |  |
| TNKS_Exon1 | rs33945943 | Arg200= | ref=G | TH_4018023001_C_AG:45 | 151 |  |
| TNKS_Exon1 | rs33945943 | Arg200= | ref=G | TH_4018024001_C_AG:41 | 174 |  |
| TNKS_Exon1 | rs33945943 | Arg200= | ref=G | TH_4018027001_C_AG:45 | 293 |  |
| TNKS_Exon1 | rs33945943 | Arg200= | ref=G | TH_418122001_C_AG:35 | 107 |  |
| TNKS_Exon1 | rs33945943 | Arg200= | ref=G |  | 108 |  |
| TNKS_Exon1 | rs33945943 | Arg200= | ref=G | TH_418124001_C_AG:50 | 129 |  |
| TNKS_Exon1 | rs33945943 | Arg200= | ref=G | TH_418125001_C_AG:50 | 185 |  |
| TNKS_Exon1 | rs33945943 | Arg200= | ref=G | TH_418136001_C_AG:50 | 164 |  |
| TNKS_Exon1 | rs33945943 | Arg200= | ref=G | TH_418148001_C_AG:31 | 169 |  |
| TNKS_Exon1 | rs33945943 | Arg200= | ref=G | TH_418159001_C_AG:38 | 145 |  |
| TNKS_Exon1 | rs33945943 | Arg200= | ref=G | TH_418160001_C_AG:42 | 110 |  |
| TNKS_Exon1 | rs33945943 | Arg200= | ref=G | TH_418167001_C_AG:41 | 119 |  |
| TNKS_Exon1 | rs33945943 | Arg200= | ref=G | TH_418168001_C_AG:47 | 146 |  |
| TNKS_Exon1 | rs33945943 | Arg200= | ref=G | TH_418171001_C_AG:48 | 219 |  |
| TNKS_Exon1 | rs33945943 | Arg200= | ref=G | TH_418300001_C_AG:45 | 313 |  |
| TNKS_Exon1 | rs33945943 | Arg200= | ref=G | TH_418352001_C_AG:50 | 157 |  |
| TNKS_Exon1 | rs33945943 | Arg200= | ref=G | TH_418360001_C_AG:44 | 180 |  |
| TNKS_Exon1 | rs33945943 | Arg200= | ref=G | TH_418683001_C_AG:45 | 212 |  |
| TNKS_Exon1 | rs33945943 | Arg200= | ref=G | TH_418701001_C_AG:38 | 124 |  |
| TNKS_Exon1 | rs33945943 | Arg200= | ref=G | TH_418748001_C_AG:46 | 296 |  |
| TNKS_Exon1 | rs34790717 | Gly237Ala | ref=G | TH_1315304001_P_AG:48 | 283 |  |
| TNKS_Exon1 | rs34790717 | Gly237Ala | ref=G | TH_1315332001_P_AG:30 | 113 |  |
| TNKS_Exon1 | rs34790717 | Gly237Ala | ref=G | TH_1415117001_P_AG:39 | 225 |  |
| TNKS_Exon1 | rs34790717 | Gly237Ala | ref=G | TH_1415120001_P_AG:49 | 275 |  |
| TNKS_Exon1 | rs34790717 | Gly237Ala | ref=G | TH_1715015001_P_AG:46 | 279 |  |
| TNKS_Exon1 | rs34790717 | Gly237Ala | ref=G | TH_1715041001_P_AG:46 | 113 |  |
| TNKS_Exon1 | rs34790717 | Gly237Ala | ref=G | TH_1715050001_P_AG:49 | 304 |  |
| TNKS_Exon1 | rs34790717 | Gly237Ala | ref=G | TH_1715053001_P_AG:42 | 251 |  |
| TNKS_Exon1 | rs34790717 | Gly237Ala | ref=G | TH_1715091001_P_AG:42 | 110 |  |
| TNKS_Exon1 | rs34790717 | Gly237Ala | ref=G | TH_1715093001_P_AG:26 | 105 |  |
| TNKS_Exon1 | rs34790717 | Gly237Ala | ref=G | TH_1715105001_P_AG:44 | 199 |  |
| TNKS_Exon1 | rs34790717 | Gly237Ala | ref=G | TH_1815079001_P_AG:48 | 273 |  |
| TNKS_Exon1 | rs34790717 | Gly237Ala | ref=G | TH_1815131001_P_AG:42 | 199 |  |
| TNKS_Exon1 | rs34790717 | Gly237Ala | ref=G | TH_1815163001_P_AG:37 | 100 |  |
| TNKS_Exon1 | rs34790717 | Gly237Ala | ref=G | TH_1815221001_P_AG:40 | 187 |  |
| TNKS_Exon1 | rs34790717 | Gly237Ala | ref=G | TH_1815247001_P_AG:35 | 255 |  |
| TNKS_Exon1 | rs34790717 | Gly237Ala | ref=G | TH_1815261001_P_AG:40 | 254 |  |
| TNKS_Exon1 | rs34790717 | Gly237Ala | ref=G | TH_1815268001_P_AG:45 | 320 |  |
| TNKS_Exon1 | rs34790717 | Gly237Ala | ref=G | TH_1815272001_P_AG:32 | 138 |  |
| TNKS_Exon1 | rs34790717 | Gly237Ala | ref=G | TH_1815284001_P_AG:47 | 298 |  |
| TNKS_Exon1 | rs34790717 | Gly237Ala | ref=G |  | 150 |  |
| TNKS_Exon1 | rs34790717 | Gly237Ala | ref=G | TH_1815293001_P_AG:35 | 243 |  |
| TNKS_Exon1 | rs34790717 | Gly237Ala | ref=G | TH_1815307001_P_AG:40 | 119 |  |
| TNKS_Exon1 | rs34790717 | Gly237Ala | ref=G | TH_1815313001_P_AG:42 | 151 |  |
| TNKS_Exon1 | rs34790717 | Gly237Ala | ref=G | TH_1815337001_P_AG:46 | 261 |  |
| TNKS_Exon1 | rs34790717 | Gly237Ala | ref=G | TH_1815484001_P_AG:44 | 315 |  |
| TNKS_Exon1 | rs34790717 | Gly237Ala | ref=G | TH_515003001_P_AG:43 | 218 |  |
| TNKS_Exon1 | rs34790717 | Gly237Ala | ref=G | TH_515015001_P_AG:39 | 216 |  |
| TNKS_Exon1 | rs34790717 | Gly237Ala | ref=G | TH_515078001_P_AG:42 | 260 |  |
| TNKS_Exon1 | rs34790717 | Gly237Ala | ref=G | TH_515091001_P_AG:40 | 299 |  |
| TNKS_Exon1 | rs34790717 | Gly237Ala | ref=G |  | 193 |  |
| TNKS_Exon1 | rs34790717 | Gly237Ala | ref=G | TH_515151001_P_AG:38 | 215 |  |
| TNKS_Exon1 | rs34790717 | Gly237Ala | ref=G | TH_515158001_P_AG:45 | 100 |  |
| TNKS_Exon1 | rs34790717 | Gly237Ala | ref=G | TH_515162001_P_AG:47 | 327 |  |
| TNKS_Exon1 | rs34790717 | Gly237Ala | ref=G | TH_515164001_P_AG:48 | 333 |  |
| TNKS_Exon1 | rs34790717 | Gly237Ala | ref=G | TH_515165001_P_AG:41 | 331 |  |
| TNKS_Exon1 | rs34790717 | Gly237Ala | ref=G | TH_515186001_P_AG:45 | 254 |  |
| TNKS_Exon1 | rs34790717 | Gly237Ala | ref=G | TH_515206001_P_AG:44 | 188 |  |
| TNKS_Exon1 | rs34790717 | Gly237Ala | ref=G | TH_515213001_P_AG:43 | 205 |  |
| TNKS_Exon1 | rs34790717 | Gly237Ala | ref=G | TH_515247001_P_AG:50 | 359 |  |
| TNKS_Exon1 | rs34790717 | Gly237Ala | ref=G | TH_515303001_P_AG:42 | 138 |  |
| TNKS_Exon1 | rs34790717 | Gly237Ala | ref=G | TH_515333001_P_AG:43 | 178 |  |
| TNKS_Exon1 | rs34790717 | Gly237Ala | ref=G | TH_715006001_P_AG:48 | 195 |  |
| TNKS_Exon1 | rs34790717 | Gly237Ala | ref=G | TH_715069001_P_AG:40 | 141 |  |
| TNKS_Exon1 | rs34790717 | Gly237Ala | ref=G | TH_715088001_P_AG:39 | 161 |  |
| TNKS_Exon1 | rs34790717 | Gly237Ala | ref=G |  | 117 |  |
| TNKS_Exon1 | rs34790717 | Gly237Ala | ref=G | TH_715184001_P_AG:33 | 243 |  |
| TNKS_Exon1 | rs34790717 | Gly237Ala | ref=G |  | 129 |  |
| TNKS_Exon1 | rs34790717 | Gly237Ala | ref=G | TH_4018014001_C_AG:36 | 111 |  |
| TNKS_Exon1 | rs34790717 | Gly237Ala | ref=G | TH_4018023001_C_AG:44 | 225 |  |
| TNKS_Exon1 | rs34790717 | Gly237Ala | ref=G | TH_4018024001_C_AG:49 | 320 |  |
| TNKS_Exon1 | rs34790717 | Gly237Ala | ref=G | TH_4018027001_C_AG:43 | 329 |  |
| TNKS_Exon1 | rs34790717 | Gly237Ala | ref=G | TH_4018051001_C_AG:50 | 295 |  |
| TNKS_Exon1 | rs34790717 | Gly237Ala | ref=G | TH_4018059001_C_AG:46 | 130 |  |
| TNKS_Exon1 | rs34790717 | Gly237Ala | ref=G | TH_4018066001_C_AG:40 | 296 |  |
| TNKS_Exon1 | rs34790717 | Gly237Ala | ref=G | TH_418122001_C_AG:40 | 228 |  |
| TNKS_Exon1 | rs34790717 | Gly237Ala | ref=G |  | 168 |  |
| TNKS_Exon1 | rs34790717 | Gly237Ala | ref=G | TH_418124001_C_AG:42 | 168 |  |
| TNKS_Exon1 | rs34790717 | Gly237Ala | ref=G | TH_418125001_C_AG:39 | 276 |  |
| TNKS_Exon1 | rs34790717 | Gly237Ala | ref=G | TH_418135001_C_AG:45 | 221 |  |
| TNKS_Exon1 | rs34790717 | Gly237Ala | ref=G | TH_418136001_C_AG:33 | 138 |  |
| TNKS_Exon1 | rs34790717 | Gly237Ala | ref=G | TH_418140001_C_AG:40 | 115 |  |
| TNKS_Exon1 | rs34790717 | Gly237Ala | ref=G | TH_418148001_C_AG:31 | 236 |  |
| TNKS_Exon1 | rs34790717 | Gly237Ala | ref=G | TH_418158001_C_AG:46 | 125 |  |
| TNKS_Exon1 | rs34790717 | Gly237Ala | ref=G | TH_418159001_C_AG:50 | 217 |  |
| TNKS_Exon1 | rs34790717 | Gly237Ala | ref=G | TH_418160001_C_AG:37 | 172 |  |
| TNKS_Exon1 | rs34790717 | Gly237Ala | ref=G | TH_418163001_C_AG:45 | 199 |  |
| TNKS_Exon1 | rs34790717 | Gly237Ala | ref=G | TH_418167001_C_AG:50 | 248 |  |
| TNKS_Exon1 | rs34790717 | Gly237Ala | ref=G | TH_418168001_C_AG:45 | 212 |  |
| TNKS_Exon1 | rs34790717 | Gly237Ala | ref=G | TH_418171001_C_AG:45 | 325 |  |
| TNKS_Exon1 | rs34790717 | Gly237Ala | ref=G | TH_418193001_C_AG:44 | 177 |  |
| TNKS_Exon1 | rs34790717 | Gly237Ala | ref=G | TH_418300001_C_AG:34 | 292 |  |
| TNKS_Exon1 | rs34790717 | Gly237Ala | ref=G | TH_418302001_C_AG:42 | 163 |  |
| TNKS_Exon1 | rs34790717 | Gly237Ala | ref=G | TH_418340001_C_AG:43 | 119 |  |
| TNKS_Exon1 | rs34790717 | Gly237Ala | ref=G | TH_418344001_C_AG:42 | 155 |  |
| TNKS_Exon1 | rs34790717 | Gly237Ala | ref=G | TH_418352001_C_AG:40 | 171 |  |
| TNKS_Exon1 | rs34790717 | Gly237Ala | ref=G | TH_418360001_C_AG:39 | 253 |  |
| TNKS_Exon1 | rs34790717 | Gly237Ala | ref=G | TH_418683001_C_AG:48 | 310 |  |
| TNKS_Exon1 | rs34790717 | Gly237Ala | ref=G | TH_418692001_C_AG:48 | 248 |  |
| TNKS_Exon1 | rs34790717 | Gly237Ala | ref=G | TH_418698001_C_AG:41 | 197 |  |
| TNKS_Exon1 | rs34790717 | Gly237Ala | ref=G | TH_418701001_C_AG:40 | 238 |  |
| TNKS_Exon1 | rs34790717 | Gly237Ala | ref=G | TH_418742001_C_AG:34 | 142 |  |
| TNKS_Exon1 | rs34790717 | Gly237Ala | ref=G | TH_418748001_C_AG:50 | 367 |  |
| TNKS_Exon11 | rs35052906 | Val573= | ref=C | hetero=TH_1315304001_P_AG:33 | score=193 |  |
| TNKS_Exon11 | rs35052906 | Val573= | ref=C | TH_1715064001_P_AG:41 | 197 |  |
| TNKS_Exon11 | rs35052906 | Val573= | ref=C | TH_1815241001_P_AG:39 | 203 |  |
| TNKS_Exon11 | rs35052906 | Val573= | ref=C | TH_1815261001_P_AG:37 | 208 |  |
| TNKS_Exon11 | rs35052906 | Val573= | ref=C | TH_1815272001_P_AG:33 | 102 |  |
| TNKS_Exon11 | rs35052906 | Val573= | ref=C | TH_1815285001_P_AG:41 | 267 |  |
| TNKS_Exon11 | rs35052906 | Val573= | ref=C | TH_1815337001_P_AG:30 | 186 |  |
| TNKS_Exon11 | rs35052906 | Val573= | ref=C | TH_1815484001_P_AG:47 | 329 |  |
| TNKS_Exon11 | rs35052906 | Val573= | ref=C | TH_515078001_P_AG:47 | 251 |  |
| TNKS_Exon11 | rs35052906 | Val573= | ref=C | TH_515091001_P_AG:30 | 183 |  |
| TNKS_Exon11 | rs35052906 | Val573= | ref=C | TH_515164001_P_AG:48 | 330 |  |
| TNKS_Exon11 | rs35052906 | Val573= | ref=C | TH_715184001_P_AG:44 | 277 |  |
| TNKS_Exon11 | rs35052906 | Val573= | ref=C | TH_4018023001_C_AG:47 | 275 |  |
| TNKS_Exon11 | rs35052906 | Val573= | ref=C | TH_4018027001_C_AG:50 | 342 |  |
| TNKS_Exon11 | rs35052906 | Val573= | ref=C | TH_4018051001_C_AG:45 | 265 |  |
| TNKS_Exon11 | rs35052906 | Val573= | ref=C | TH_418123001_C_AG:46 | 284 |  |
| TNKS_Exon11 | rs35052906 | Val573= | ref=C | TH_418124001_C_AG:32 | 136 |  |
| TNKS_Exon11 | rs35052906 | Val573= | ref=C | TH_418135001_C_AG:46 | 227 |  |
| TNKS_Exon11 | rs35052906 | Val573= | ref=C | TH_418136001_C_AG:45 | 192 |  |
| TNKS_Exon11 | rs35052906 | Val573= | ref=C | TH_418148001_C_AG:17 | 102 |  |
| TNKS_Exon11 | rs35052906 | Val573= | ref=C | TH_418163001_C_AG:35 | 111 |  |
| TNKS_Exon11 | rs35052906 | Val573= | ref=C | TH_418193001_C_AG:35 | 111 |  |
| TNKS_Exon11 | rs35052906 | Val573= | ref=C | TH_418360001_C_AG:44 | 224 |  |
| TNKS_Exon11 | rs35052906 | Val573= | ref=C | TH_418692001_C_AG:34 | 135 |  |
| TNKS_Exon11 | rs35052906 | Val573= | ref=C | TH_418698001_C_AG:34 | 162 |  |
| TNKS_Exon12 | rs6601360 | Gly602= | ref=T |  | 105 |  |
| TNKS_Exon12 | rs6601360 | Gly602= | ref=T |  | 150 |  |
| TNKS_Exon12 | rs6601360 | Gly602= | ref=T |  | 141 |  |
| TNKS_Exon12 | rs6601360 | Gly602= | ref=T |  | 102 |  |
| TNKS_Exon12 | rs6601360 | Gly602= | ref=T |  | 123 |  |
| TNKS_Exon12 | rs6601360 | Gly602= | ref=T |  | 126 |  |
| TNKS_Exon12 | rs6601360 | Gly602= | ref=T | hetero=TH_1415121001_P_AG:44 | 252 |  |
| TNKS_Exon12 | rs6601360 | Gly602= | ref=T |  | 334 |  |
| TNKS_Exon12 | rs6601360 | Gly602= | ref=T |  | 184 |  |
| TNKS_Exon12 | rs6601360 | Gly602= | ref=T |  | 102 |  |
| TNKS_Exon12 | rs6601360 | Gly602= | ref=T |  | 141 |  |
| TNKS_Exon12 | rs6601360 | Gly602= | ref=T |  | 117 |  |
| TNKS_Exon12 | rs6601360 | Gly602= | ref=T |  | 214 |  |
| TNKS_Exon12 | rs6601360 | Gly602= | ref=T |  | 159 |  |
| TNKS_Exon12 | rs6601360 | Gly602= | ref=T |  | 168 |  |
| TNKS_Exon12 | rs6601360 | Gly602= | ref=T |  | 238 |  |
| TNKS_Exon12 | rs6601360 | Gly602= | ref=T |  | 153 |  |
| TNKS_Exon12 | rs6601360 | Gly602= | ref=T |  | 132 |  |
| TNKS_Exon12 | rs6601360 | Gly602= | ref=T |  | 105 |  |
| TNKS_Exon12 | rs6601360 | Gly602= | ref=T |  | 117 |  |
| TNKS_Exon12 | rs6601360 | Gly602= | ref=T |  | 108 |  |
| TNKS_Exon12 | rs6601360 | Gly602= | ref=T |  | 111 |  |
| TNKS_Exon12 | rs6601360 | Gly602= | ref=T |  | 111 |  |
| TNKS_Exon12 | rs6601360 | Gly602= | ref=T |  | 135 |  |
| TNKS_Exon12 | rs6601360 | Gly602= | ref=T |  | 138 |  |
| TNKS_Exon12 | rs6601360 | Gly602= | ref=T |  | 108 |  |
| TNKS_Exon12 | rs6601360 | Gly602= | ref=T |  | 108 |  |
| TNKS_Exon12 | rs6601360 | Gly602= | ref=T |  | 129 |  |
| TNKS_Exon12 | rs6601360 | Gly602= | ref=T |  | 165 |  |
| TNKS_Exon12 | rs6601360 | Gly602= | ref=T |  | 123 |  |
| TNKS_Exon12 | rs6601360 | Gly602= | ref=T |  | 193 |  |
| TNKS_Exon12 | rs6601360 | Gly602= | ref=T |  | 114 |  |
| TNKS_Exon12 | rs6601360 | Gly602= | ref=T |  | 114 |  |
| TNKS_Exon12 | rs6601360 | Gly602= | ref=T |  | 118 |  |
| TNKS_Exon12 | rs6601360 | Gly602= | ref=T |  | 150 |  |
| TNKS_Exon12 | rs6601360 | Gly602= | ref=T |  | 181 |  |
| TNKS_Exon12 | rs6601360 | Gly602= | ref=T |  | 117 |  |
| TNKS_Exon12 | rs6601360 | Gly602= | ref=T |  | 126 |  |
| TNKS_Exon12 | rs6601360 | Gly602= | ref=T |  | 134 |  |
| TNKS_Exon12 | rs6601360 | Gly602= | ref=T |  | 147 |  |
| TNKS_Exon12 | rs6601360 | Gly602= | ref=T |  | 141 |  |
| TNKS_Exon12 | rs6601360 | Gly602= | ref=T |  | 120 |  |
| TNKS_Exon12 | rs6601360 | Gly602= | ref=T |  | 129 |  |
| TNKS_Exon12 | rs6601360 | Gly602= | ref=T |  | 238 |  |
| TNKS_Exon12 | rs6601360 | Gly602= | ref=T |  | 171 |  |
| TNKS_Exon12 | rs6601360 | Gly602= | ref=T |  | 104 |  |
| TNKS_Exon12 | rs6601360 | Gly602= | ref=T |  | 132 |  |
| TNKS_Exon12 | rs6601360 | Gly602= | ref=T |  | 132 |  |
| TNKS_Exon12 | rs6601360 | Gly602= | ref=T |  | 165 |  |
| TNKS_Exon12 | rs6601360 | Gly602= | ref=T |  | 190 |  |
| TNKS_Exon12 | rs6601360 | Gly602= | ref=T |  | 150 |  |
| TNKS_Exon12 | rs6601360 | Gly602= | ref=T |  | 153 |  |
| TNKS_Exon12 | rs6601360 | Gly602= | ref=T |  | 126 |  |
| TNKS_Exon12 | rs6601360 | Gly602= | ref=T |  | 205 |  |
| TNKS_Exon12 | rs6601360 | Gly602= | ref=T |  | 102 |  |
| TNKS_Exon12 | rs6601360 | Gly602= | ref=T |  | 187 |  |
| TNKS_Exon12 | rs6601360 | Gly602= | ref=T |  | 214 |  |
| TNKS_Exon12 | rs6601360 | Gly602= | ref=T |  | 298 |  |
| TNKS_Exon12 | rs6601360 | Gly602= | ref=T |  | 105 |  |
| TNKS_Exon12 | rs6601360 | Gly602= | ref=T |  | 144 |  |
| TNKS_Exon12 | rs6601360 | Gly602= | ref=T |  | 144 |  |
| TNKS_Exon12 | rs6601360 | Gly602= | ref=T |  | 105 |  |
| TNKS_Exon12 | rs6601360 | Gly602= | ref=T |  | 114 |  |
| TNKS_Exon12 | rs6601360 | Gly602= | ref=T |  | 102 |  |
| TNKS_Exon12 | rs6601360 | Gly602= | ref=T |  | 105 |  |
| TNKS_Exon12 | rs6601360 | Gly602= | ref=T |  | 193 |  |
| TNKS_Exon12 | rs6601360 | Gly602= | ref=T |  | 144 |  |
| TNKS_Exon12 | rs6601360 | Gly602= | ref=T |  | 135 |  |
| TNKS_Exon12 | rs6601360 | Gly602= | ref=T |  | 117 |  |
| TNKS_Exon12 | rs6601360 | Gly602= | ref=T |  | 313 |  |
| TNKS_Exon12 | rs6601360 | Gly602= | ref=T |  | 174 |  |
| TNKS_Exon12 | rs6601360 | Gly602= | ref=T |  | 199 |  |
| TNKS_Exon12 | rs6601360 | Gly602= | ref=T |  | 412 |  |
| TNKS_Exon12 | rs6601360 | Gly602= | ref=T |  | 120 |  |
| TNKS_Exon12 | rs6601360 | Gly602= | ref=T |  | 111 |  |
| TNKS_Exon12 | rs6601360 | Gly602= | ref=T |  | 102 |  |
| TNKS_Exon12 | rs6601360 | Gly602= | ref=T |  | 141 |  |
| TNKS_Exon12 | rs6601360 | Gly602= | ref=T |  | 123 |  |
| TNKS_Exon12 | rs6601360 | Gly602= | ref=T |  | 108 |  |
| TNKS_Exon12 | rs6601360 | Gly602= | ref=T |  | 208 |  |
| TNKS_Exon12 | rs6601360 | Gly602= | ref=T |  | 138 |  |
| TNKS_Exon12 | rs6601360 | Gly602= | ref=T |  | 149 |  |
| TNKS_Exon12 | rs6601360 | Gly602= | ref=T |  | 415 |  |
| TNKS_Exon12 | rs6601360 | Gly602= | ref=T |  | 246 |  |
| TNKS_Exon12 | rs6601360 | Gly602= | ref=T |  | 129 |  |
| TNKS_Exon12 | rs6601360 | Gly602= | ref=T |  | 346 |  |
| TNKS_Exon12 | rs6601360 | Gly602= | ref=T |  | 111 |  |
| TNKS_Exon12 | rs6601360 | Gly602= | ref=T |  | 132 |  |
| TNKS_Exon12 | rs6601360 | Gly602= | ref=T |  | 129 |  |
| TNKS_Exon12 | rs6601360 | Gly602= | ref=T |  | 119 |  |
| TNKS_Exon12 | rs6601360 | Gly602= | ref=T |  | 129 |  |
| TNKS_Exon12 | rs6601360 | Gly602= | ref=T |  | 153 |  |
| TNKS_Exon12 | rs6601360 | Gly602= | ref=T |  | 171 |  |
| TNKS_Exon12 | rs6601360 | Gly602= | ref=T |  | 147 |  |
| TNKS_Exon12 | rs6601360 | Gly602= | ref=T |  | 114 |  |
| TNKS_Exon12 | rs6601360 | Gly602= | ref=T |  | 102 |  |
| TNKS_Exon12 | rs6601360 | Gly602= | ref=T |  | 102 |  |
| TNKS_Exon12 | rs6601360 | Gly602= | ref=T |  | 123 |  |
| TNKS_Exon12 | rs6601360 | Gly602= | ref=T |  | 105 |  |
| TNKS_Exon12 | rs6601360 | Gly602= | ref=T |  | 108 |  |
| TNKS_Exon12 | rs6601360 | Gly602= | ref=T |  | 138 |  |
| TNKS_Exon12 | rs6601360 | Gly602= | ref=T |  | 171 |  |
| TNKS_Exon12 | rs6601360 | Gly602= | ref=T |  | 111 |  |
| TNKS_Exon12 | rs6601360 | Gly602= | ref=T |  | 102 |  |
| TNKS_Exon12 | rs6601360 | Gly602= | ref=T |  | 103 |  |
| TNKS_Exon12 | rs6601360 | Gly602= | ref=T |  | 168 |  |
| TNKS_Exon12 | rs6601360 | Gly602= | ref=T |  | 102 |  |
| TNKS_Exon12 | rs6601360 | Gly602= | ref=T |  | 105 |  |
| TNKS_Exon12 | rs6601360 | Gly602= | ref=T |  | 574 |  |
| TNKS_Exon12 | rs6601360 | Gly602= | ref=T |  | 161 |  |
| TNKS_Exon12 | rs6601360 | Gly602= | ref=T |  | 102 |  |
| TNKS_Exon12 | rs6601360 | Gly602= | ref=T |  | 105 |  |
| TNKS_Exon12 | rs6601360 | Gly602= | ref=T |  | 120 |  |
| TNKS_Exon12 | rs6601360 | Gly602= | ref=T |  | 126 |  |
| TNKS_Exon12 | rs6601360 | Gly602= | ref=T |  | 147 |  |
| TNKS_Exon12 | rs6601360 | Gly602= | ref=T |  | 105 |  |
| TNKS_Exon12 | rs6601360 | Gly602= | ref=T |  | 122 |  |
| TNKS_Exon12 | rs6601360 | Gly602= | ref=T |  | 123 |  |
| TNKS_Exon12 | rs6601360 | Gly602= | ref=T |  | 428 |  |
| TNKS_Exon16 | rs61752022 | Thr777= | ref=T | hetero=TH_515074001_P_AG:50 | score=166 |  |
| TNKS_Exon16 | rs61752022 | Thr777= | ref=T | hetero=TH_515294001_P_AG:43 | score=232 |  |
| TNKS_Exon16 | rs61752022 | Thr777= | ref=T | hetero=TH_715092001_P_AG:46 | score=118 |  |
| TNKS_Exon16 | rs61752022 | Thr777= | ref=T | hetero=TH_418748001_C_AG:46 | score=332 |  |
| TNKS_Exon19 | rs13265931 | Ala1015= | ref=G | TH_1315332001_P_AG:45 | 187 |  |
| TNKS_Exon19 | rs13265931 | Ala1015= | ref=G | TH_1315347001_P_AG:35 | 169 |  |
| TNKS_Exon19 | rs13265931 | Ala1015= | ref=G | TH_1315376001_P_AG:42 | 172 |  |
| TNKS_Exon19 | rs13265931 | Ala1015= | ref=G | TH_1415117001_P_AG:46 | 218 |  |
| TNKS_Exon19 | rs13265931 | Ala1015= | ref=G | TH_1415120001_P_AG:43 | 247 |  |
| TNKS_Exon19 | rs13265931 | Ala1015= | ref=G | TH_1415121001_P_AG:50 | 273 |  |
| TNKS_Exon19 | rs13265931 | Ala1015= | ref=G | TH_1715015001_P_AG:36 | 174 |  |
| TNKS_Exon19 | rs13265931 | Ala1015= | ref=G | TH_1715050001_P_AG:44 | 252 |  |
| TNKS_Exon19 | rs13265931 | Ala1015= | ref=G | TH_1715053001_P_AG:42 | 246 |  |
| TNKS_Exon19 | rs13265931 | Ala1015= | ref=G | TH_1715064001_P_AG:44 | 115 |  |
| TNKS_Exon19 | rs13265931 | Ala1015= | ref=G | TH_1715091001_P_AG:50 | 127 |  |
| TNKS_Exon19 | rs13265931 | Ala1015= | ref=G | TH_1715101001_P_AG:40 | 156 |  |
| TNKS_Exon19 | rs13265931 | Ala1015= | ref=G | TH_1815079001_P_AG:37 | 212 |  |
| TNKS_Exon19 | rs13265931 | Ala1015= | ref=G | TH_1815131001_P_AG:31 | 100 |  |
| TNKS_Exon19 | rs13265931 | Ala1015= | ref=G | TH_1815221001_P_AG:38 | 135 |  |
| TNKS_Exon19 | rs13265931 | Ala1015= | ref=G |  | 162 |  |
| TNKS_Exon19 | rs13265931 | Ala1015= | ref=G | TH_1815268001_P_AG:36 | 258 |  |
| TNKS_Exon19 | rs13265931 | Ala1015= | ref=G | TH_1815284001_P_AG:28 | 147 |  |
| TNKS_Exon19 | rs13265931 | Ala1015= | ref=G | TH_1815285001_P_AG:39 | 189 |  |
| TNKS_Exon19 | rs13265931 | Ala1015= | ref=G | TH_1815293001_P_AG:37 | 231 |  |
| TNKS_Exon19 | rs13265931 | Ala1015= | ref=G | TH_1815313001_P_AG:44 | 180 |  |
| TNKS_Exon19 | rs13265931 | Ala1015= | ref=G | TH_1815410001_P_AG:40 | 107 |  |
| TNKS_Exon19 | rs13265931 | Ala1015= | ref=G | TH_515003001_P_AG:50 | 140 |  |
| TNKS_Exon19 | rs13265931 | Ala1015= | ref=G | TH_515015001_P_AG:46 | 234 |  |
| TNKS_Exon19 | rs13265931 | Ala1015= | ref=G | TH_515035001_P_AG:43 | 212 |  |
| TNKS_Exon19 | rs13265931 | Ala1015= | ref=G | TH_515037001_P_AG:45 | 321 |  |
| TNKS_Exon19 | rs13265931 | Ala1015= | ref=G | TH_515046001_P_AG:40 | 115 |  |
| TNKS_Exon19 | rs13265931 | Ala1015= | ref=G | TH_515080001_P_AG:43 | 204 |  |
| TNKS_Exon19 | rs13265931 | Ala1015= | ref=G |  | 165 |  |
| TNKS_Exon19 | rs13265931 | Ala1015= | ref=G | TH_515151001_P_AG:35 | 179 |  |
| TNKS_Exon19 | rs13265931 | Ala1015= | ref=G | TH_515162001_P_AG:49 | 317 |  |
| TNKS_Exon19 | rs13265931 | Ala1015= | ref=G | TH_515165001_P_AG:49 | 347 |  |
| TNKS_Exon19 | rs13265931 | Ala1015= | ref=G | TH_515175001_P_AG:35 | 110 |  |
| TNKS_Exon19 | rs13265931 | Ala1015= | ref=G | TH_515178001_P_AG:45 | 186 |  |
| TNKS_Exon19 | rs13265931 | Ala1015= | ref=G | TH_515186001_P_AG:43 | 208 |  |
| TNKS_Exon19 | rs13265931 | Ala1015= | ref=G | TH_515206001_P_AG:30 | 123 |  |
| TNKS_Exon19 | rs13265931 | Ala1015= | ref=G | TH_515211001_P_AG:50 | 200 |  |
| TNKS_Exon19 | rs13265931 | Ala1015= | ref=G | TH_515213001_P_AG:47 | 272 |  |
| TNKS_Exon19 | rs13265931 | Ala1015= | ref=G | TH_515247001_P_AG:47 | 343 |  |
| TNKS_Exon19 | rs13265931 | Ala1015= | ref=G | TH_515303001_P_AG:40 | 114 |  |
| TNKS_Exon19 | rs13265931 | Ala1015= | ref=G | TH_515333001_P_AG:46 | 109 |  |
| TNKS_Exon19 | rs13265931 | Ala1015= | ref=G | TH_715069001_P_AG:33 | 149 |  |
| TNKS_Exon19 | rs13265931 | Ala1015= | ref=G | TH_4018014001_C_AG:45 | 156 |  |
| TNKS_Exon19 | rs13265931 | Ala1015= | ref=G | TH_4018059001_C_AG:47 | 148 |  |
| TNKS_Exon19 | rs13265931 | Ala1015= | ref=G | TH_418122001_C_AG:37 | 156 |  |
| TNKS_Exon19 | rs13265931 | Ala1015= | ref=G | TH_418123001_C_AG:45 | 246 |  |
| TNKS_Exon19 | rs13265931 | Ala1015= | ref=G | TH_418125001_C_AG:46 | 232 |  |
| TNKS_Exon19 | rs13265931 | Ala1015= | ref=G | TH_418140001_C_AG:45 | 203 |  |
| TNKS_Exon19 | rs13265931 | Ala1015= | ref=G | TH_418141001_C_AG:42 | 125 |  |
| TNKS_Exon19 | rs13265931 | Ala1015= | ref=G | TH_418156001_C_AG:46 | 216 |  |
| TNKS_Exon19 | rs13265931 | Ala1015= | ref=G | TH_418159001_C_AG:50 | 139 |  |
| TNKS_Exon19 | rs13265931 | Ala1015= | ref=G |  | 108 |  |
| TNKS_Exon19 | rs13265931 | Ala1015= | ref=G | TH_418167001_C_AG:47 | 244 |  |
| TNKS_Exon19 | rs13265931 | Ala1015= | ref=G | TH_418168001_C_AG:37 | 104 |  |
| TNKS_Exon19 | rs13265931 | Ala1015= | ref=G | TH_418171001_C_AG:49 | 331 |  |
| TNKS_Exon19 | rs13265931 | Ala1015= | ref=G | TH_418300001_C_AG:48 | 359 |  |
| TNKS_Exon19 | rs13265931 | Ala1015= | ref=G | TH_418302001_C_AG:40 | 137 |  |
| TNKS_Exon19 | rs13265931 | Ala1015= | ref=G | TH_418340001_C_AG:50 | 152 |  |
| TNKS_Exon19 | rs13265931 | Ala1015= | ref=G | TH_418344001_C_AG:50 | 106 |  |
| TNKS_Exon2 |  | Pro275Ala | ref=C | hetero=TH_1715050001_P_AG:48 | score=336 |  |
| TNKS_Exon22 |  | Asn1103= | ref=T | hetero=TH_418319001_C_AG:36 | score=131 |  |
| TNKS_Exon26 |  | His1270= | ref=C | hetero=TH_1415154001_P_AG:42 | score=163 |  |
| TNKS_Exon7 |  | Val417= | ref=C | hetero=TH_1715048001_P_AG:48 | score=367 |  |
| TNKS_Exon8 | rs7006985 | Thr462= | ref=A | hetero=TH_1315304001_P_AG:48 | 202 |  |
| TNKS_Exon8 | rs7006985 | Thr462= | ref=A | TH_1315332001_P_AG:36 | 148 |  |
| TNKS_Exon8 | rs7006985 | Thr462= | ref=A |  | 181 |  |
| TNKS_Exon8 | rs7006985 | Thr462= | ref=A | TH_1315347001_P_AG:39 | 208 |  |
| TNKS_Exon8 | rs7006985 | Thr462= | ref=A | TH_1315376001_P_AG:42 | 125 |  |
| TNKS_Exon8 | rs7006985 | Thr462= | ref=A | TH_1415112001_P_AG:39 | 151 |  |
| TNKS_Exon8 | rs7006985 | Thr462= | ref=A |  | 108 |  |
| TNKS_Exon8 | rs7006985 | Thr462= | ref=A | TH_1415116001_P_AG:42 | 104 |  |
| TNKS_Exon8 | rs7006985 | Thr462= | ref=A | TH_1415117001_P_AG:40 | 230 |  |
| TNKS_Exon8 | rs7006985 | Thr462= | ref=A | TH_1415121001_P_AG:41 | 279 |  |
| TNKS_Exon8 | rs7006985 | Thr462= | ref=A |  | 328 |  |
| TNKS_Exon8 | rs7006985 | Thr462= | ref=A |  | 241 |  |
| TNKS_Exon8 | rs7006985 | Thr462= | ref=A | TH_1715005001_P_AG:44 | 126 |  |
| TNKS_Exon8 | rs7006985 | Thr462= | ref=A |  | 108 |  |
| TNKS_Exon8 | rs7006985 | Thr462= | ref=A |  | 217 |  |
| TNKS_Exon8 | rs7006985 | Thr462= | ref=A |  | 196 |  |
| TNKS_Exon8 | rs7006985 | Thr462= | ref=A | TH_1715029001_P_AG:47 | 148 |  |
| TNKS_Exon8 | rs7006985 | Thr462= | ref=A | TH_1715034001_P_AG:34 | 270 |  |
| TNKS_Exon8 | rs7006985 | Thr462= | ref=A | TH_1715040001_P_AG:50 | 159 |  |
| TNKS_Exon8 | rs7006985 | Thr462= | ref=A |  | 277 |  |
| TNKS_Exon8 | rs7006985 | Thr462= | ref=A | TH_1715050001_P_AG:48 | 280 |  |
| TNKS_Exon8 | rs7006985 | Thr462= | ref=A |  | 123 |  |
| TNKS_Exon8 | rs7006985 | Thr462= | ref=A | TH_1715053001_P_AG:45 | 252 |  |
| TNKS_Exon8 | rs7006985 | Thr462= | ref=A | TH_1715054001_P_AG:47 | 177 |  |
| TNKS_Exon8 | rs7006985 | Thr462= | ref=A |  | 120 |  |
| TNKS_Exon8 | rs7006985 | Thr462= | ref=A |  | 105 |  |
| TNKS_Exon8 | rs7006985 | Thr462= | ref=A | TH_1715091001_P_AG:45 | 100 |  |
| TNKS_Exon8 | rs7006985 | Thr462= | ref=A | TH_1715093001_P_AG:45 | 166 |  |
| TNKS_Exon8 | rs7006985 | Thr462= | ref=A |  | 120 |  |
| TNKS_Exon8 | rs7006985 | Thr462= | ref=A |  | 111 |  |
| TNKS_Exon8 | rs7006985 | Thr462= | ref=A | TH_1815079001_P_AG:40 | 224 |  |
| TNKS_Exon8 | rs7006985 | Thr462= | ref=A |  | 138 |  |
| TNKS_Exon8 | rs7006985 | Thr462= | ref=A | TH_1815131001_P_AG:46 | 247 |  |
| TNKS_Exon8 | rs7006985 | Thr462= | ref=A | TH_1815163001_P_AG:50 | 147 |  |
| TNKS_Exon8 | rs7006985 | Thr462= | ref=A | TH_1815221001_P_AG:42 | 139 |  |
| TNKS_Exon8 | rs7006985 | Thr462= | ref=A | TH_1815241001_P_AG:45 | 251 |  |
| TNKS_Exon8 | rs7006985 | Thr462= | ref=A |  | 165 |  |
| TNKS_Exon8 | rs7006985 | Thr462= | ref=A | TH_1815261001_P_AG:38 | 232 |  |
| TNKS_Exon8 | rs7006985 | Thr462= | ref=A | TH_1815268001_P_AG:50 | 334 |  |
| TNKS_Exon8 | rs7006985 | Thr462= | ref=A | TH_1815278001_P_AG:36 | 241 |  |
| TNKS_Exon8 | rs7006985 | Thr462= | ref=A | TH_1815284001_P_AG:46 | 301 |  |
| TNKS_Exon8 | rs7006985 | Thr462= | ref=A | TH_1815293001_P_AG:44 | 311 |  |
| TNKS_Exon8 | rs7006985 | Thr462= | ref=A | TH_1815307001_P_AG:45 | 167 |  |
| TNKS_Exon8 | rs7006985 | Thr462= | ref=A | TH_1815337001_P_AG:48 | 246 |  |
| TNKS_Exon8 | rs7006985 | Thr462= | ref=A |  | 162 |  |
| TNKS_Exon8 | rs7006985 | Thr462= | ref=A |  | 138 |  |
| TNKS_Exon8 | rs7006985 | Thr462= | ref=A | TH_1815484001_P_AG:46 | 301 |  |
| TNKS_Exon8 | rs7006985 | Thr462= | ref=A | TH_515015001_P_AG:48 | 255 |  |
| TNKS_Exon8 | rs7006985 | Thr462= | ref=A | TH_515016001_P_AG:38 | 153 |  |
| TNKS_Exon8 | rs7006985 | Thr462= | ref=A | TH_515018001_P_AG:34 | 175 |  |
| TNKS_Exon8 | rs7006985 | Thr462= | ref=A | TH_515035001_P_AG:47 | 269 |  |
| TNKS_Exon8 | rs7006985 | Thr462= | ref=A | TH_515037001_P_AG:46 | 338 |  |
| TNKS_Exon8 | rs7006985 | Thr462= | ref=A | TH_515039001_P_AG:36 | 250 |  |
| TNKS_Exon8 | rs7006985 | Thr462= | ref=A | TH_515046001_P_AG:36 | 148 |  |
| TNKS_Exon8 | rs7006985 | Thr462= | ref=A | TH_515062001_P_AG:43 | 184 |  |
| TNKS_Exon8 | rs7006985 | Thr462= | ref=A | TH_515074001_P_AG:48 | 284 |  |
| TNKS_Exon8 | rs7006985 | Thr462= | ref=A | TH_515078001_P_AG:37 | 204 |  |
| TNKS_Exon8 | rs7006985 | Thr462= | ref=A | TH_515091001_P_AG:41 | 241 |  |
| TNKS_Exon8 | rs7006985 | Thr462= | ref=A |  | 206 |  |
| TNKS_Exon8 | rs7006985 | Thr462= | ref=A |  | 102 |  |
| TNKS_Exon8 | rs7006985 | Thr462= | ref=A |  | 153 |  |
| TNKS_Exon8 | rs7006985 | Thr462= | ref=A | TH_515112001_P_AG:39 | 278 |  |
| TNKS_Exon8 | rs7006985 | Thr462= | ref=A |  | 171 |  |
| TNKS_Exon8 | rs7006985 | Thr462= | ref=A | TH_515151001_P_AG:45 | 260 |  |
| TNKS_Exon8 | rs7006985 | Thr462= | ref=A | TH_515162001_P_AG:49 | 320 |  |
| TNKS_Exon8 | rs7006985 | Thr462= | ref=A | TH_515164001_P_AG:49 | 327 |  |
| TNKS_Exon8 | rs7006985 | Thr462= | ref=A | TH_515165001_P_AG:44 | 345 |  |
| TNKS_Exon8 | rs7006985 | Thr462= | ref=A | TH_515167001_P_AG:47 | 180 |  |
| TNKS_Exon8 | rs7006985 | Thr462= | ref=A | TH_515178001_P_AG:41 | 214 |  |
| TNKS_Exon8 | rs7006985 | Thr462= | ref=A | TH_515186001_P_AG:42 | 233 |  |
| TNKS_Exon8 | rs7006985 | Thr462= | ref=A |  | 156 |  |
| TNKS_Exon8 | rs7006985 | Thr462= | ref=A |  | 193 |  |
| TNKS_Exon8 | rs7006985 | Thr462= | ref=A | TH_515201001_P_AG:34 | 157 |  |
| TNKS_Exon8 | rs7006985 | Thr462= | ref=A |  | 126 |  |
| TNKS_Exon8 | rs7006985 | Thr462= | ref=A | TH_515206001_P_AG:43 | 170 |  |
| TNKS_Exon8 | rs7006985 | Thr462= | ref=A | TH_515211001_P_AG:45 | 190 |  |
| TNKS_Exon8 | rs7006985 | Thr462= | ref=A | TH_515215001_P_AG:44 | 320 |  |
| TNKS_Exon8 | rs7006985 | Thr462= | ref=A |  | 153 |  |
| TNKS_Exon8 | rs7006985 | Thr462= | ref=A | TH_515223001_P_AG:50 | 232 |  |
| TNKS_Exon8 | rs7006985 | Thr462= | ref=A | TH_515247001_P_AG:50 | 343 |  |
| TNKS_Exon8 | rs7006985 | Thr462= | ref=A | TH_515277001_P_AG:44 | 162 |  |
| TNKS_Exon8 | rs7006985 | Thr462= | ref=A | TH_515280001_P_AG:43 | 135 |  |
| TNKS_Exon8 | rs7006985 | Thr462= | ref=A | TH_515285001_P_AG:43 | 147 |  |
| TNKS_Exon8 | rs7006985 | Thr462= | ref=A | TH_515293001_P_AG:47 | 324 |  |
| TNKS_Exon8 | rs7006985 | Thr462= | ref=A | TH_515294001_P_AG:43 | 324 |  |
| TNKS_Exon8 | rs7006985 | Thr462= | ref=A |  | 105 |  |
| TNKS_Exon8 | rs7006985 | Thr462= | ref=A | TH_515308001_P_AG:30 | 109 |  |
| TNKS_Exon8 | rs7006985 | Thr462= | ref=A | TH_515333001_P_AG:42 | 156 |  |
| TNKS_Exon8 | rs7006985 | Thr462= | ref=A |  | 114 |  |
| TNKS_Exon8 | rs7006985 | Thr462= | ref=A | TH_715069001_P_AG:47 | 163 |  |
| TNKS_Exon8 | rs7006985 | Thr462= | ref=A |  | 135 |  |
| TNKS_Exon8 | rs7006985 | Thr462= | ref=A | TH_715088001_P_AG:42 | 122 |  |
| TNKS_Exon8 | rs7006985 | Thr462= | ref=A | TH_715089001_P_AG:42 | 179 |  |
| TNKS_Exon8 | rs7006985 | Thr462= | ref=A | TH_715092001_P_AG:48 | 218 |  |
| TNKS_Exon8 | rs7006985 | Thr462= | ref=A | TH_715184001_P_AG:39 | 264 |  |
| TNKS_Exon8 | rs7006985 | Thr462= | ref=A |  | 153 |  |
| TNKS_Exon8 | rs7006985 | Thr462= | ref=A |  | 108 |  |
| TNKS_Exon8 | rs7006985 | Thr462= | ref=A | TH_4018006001_C_AG:47 | 248 |  |
| TNKS_Exon8 | rs7006985 | Thr462= | ref=A |  | 196 |  |
| TNKS_Exon8 | rs7006985 | Thr462= | ref=A |  | 156 |  |
| TNKS_Exon8 | rs7006985 | Thr462= | ref=A | TH_4018014001_C_AG:45 | 176 |  |
| TNKS_Exon8 | rs7006985 | Thr462= | ref=A |  | 105 |  |
| TNKS_Exon8 | rs7006985 | Thr462= | ref=A |  | 120 |  |
| TNKS_Exon8 | rs7006985 | Thr462= | ref=A | TH_4018023001_C_AG:48 | 267 |  |
| TNKS_Exon8 | rs7006985 | Thr462= | ref=A | TH_4018024001_C_AG:44 | 326 |  |
| TNKS_Exon8 | rs7006985 | Thr462= | ref=A | TH_4018027001_C_AG:47 | 350 |  |
| TNKS_Exon8 | rs7006985 | Thr462= | ref=A |  | 286 |  |
| TNKS_Exon8 | rs7006985 | Thr462= | ref=A | TH_4018051001_C_AG:44 | 241 |  |
| TNKS_Exon8 | rs7006985 | Thr462= | ref=A |  | 111 |  |
| TNKS_Exon8 | rs7006985 | Thr462= | ref=A | TH_4018057001_C_AG:38 | 138 |  |
| TNKS_Exon8 | rs7006985 | Thr462= | ref=A | TH_4018059001_C_AG:45 | 166 |  |
| TNKS_Exon8 | rs7006985 | Thr462= | ref=A | TH_4018060001_C_AG:50 | 114 |  |
| TNKS_Exon8 | rs7006985 | Thr462= | ref=A |  | 397 |  |
| TNKS_Exon8 | rs7006985 | Thr462= | ref=A |  | 108 |  |
| TNKS_Exon8 | rs7006985 | Thr462= | ref=A |  | 156 |  |
| TNKS_Exon8 | rs7006985 | Thr462= | ref=A |  | 102 |  |
| TNKS_Exon8 | rs7006985 | Thr462= | ref=A | TH_418122001_C_AG:47 | 258 |  |
| TNKS_Exon8 | rs7006985 | Thr462= | ref=A | TH_418125001_C_AG:47 | 247 |  |
| TNKS_Exon8 | rs7006985 | Thr462= | ref=A | TH_418127001_C_AG:36 | 238 |  |
| TNKS_Exon8 | rs7006985 | Thr462= | ref=A | TH_418133001_C_AG:42 | 208 |  |
| TNKS_Exon8 | rs7006985 | Thr462= | ref=A | TH_418135001_C_AG:43 | 177 |  |
| TNKS_Exon8 | rs7006985 | Thr462= | ref=A | TH_418136001_C_AG:46 | 227 |  |
| TNKS_Exon8 | rs7006985 | Thr462= | ref=A |  | 117 |  |
| TNKS_Exon8 | rs7006985 | Thr462= | ref=A | TH_418140001_C_AG:26 | 101 |  |
| TNKS_Exon8 | rs7006985 | Thr462= | ref=A | TH_418141001_C_AG:31 | 105 |  |
| TNKS_Exon8 | rs7006985 | Thr462= | ref=A | TH_418142001_C_AG:44 | 229 |  |
| TNKS_Exon8 | rs7006985 | Thr462= | ref=A | TH_418144001_C_AG:42 | 183 |  |
| TNKS_Exon8 | rs7006985 | Thr462= | ref=A |  | 111 |  |
| TNKS_Exon8 | rs7006985 | Thr462= | ref=A | TH_418148001_C_AG:20 | 119 |  |
| TNKS_Exon8 | rs7006985 | Thr462= | ref=A | TH_418156001_C_AG:40 | 149 |  |
| TNKS_Exon8 | rs7006985 | Thr462= | ref=A | TH_418158001_C_AG:38 | 142 |  |
| TNKS_Exon8 | rs7006985 | Thr462= | ref=A | TH_418159001_C_AG:31 | 105 |  |
| TNKS_Exon8 | rs7006985 | Thr462= | ref=A |  | 126 |  |
| TNKS_Exon8 | rs7006985 | Thr462= | ref=A | TH_418165001_C_AG:50 | 256 |  |
| TNKS_Exon8 | rs7006985 | Thr462= | ref=A | TH_418167001_C_AG:45 | 225 |  |
| TNKS_Exon8 | rs7006985 | Thr462= | ref=A | TH_418168001_C_AG:29 | 123 |  |
| TNKS_Exon8 | rs7006985 | Thr462= | ref=A |  | 111 |  |
| TNKS_Exon8 | rs7006985 | Thr462= | ref=A |  | 147 |  |
| TNKS_Exon8 | rs7006985 | Thr462= | ref=A | TH_418171001_C_AG:31 | 232 |  |
| TNKS_Exon8 | rs7006985 | Thr462= | ref=A | TH_418175001_C_AG:40 | 154 |  |
| TNKS_Exon8 | rs7006985 | Thr462= | ref=A |  | 105 |  |
| TNKS_Exon8 | rs7006985 | Thr462= | ref=A |  | 114 |  |
| TNKS_Exon8 | rs7006985 | Thr462= | ref=A | TH_418193001_C_AG:42 | 120 |  |
| TNKS_Exon8 | rs7006985 | Thr462= | ref=A | TH_418196001_C_AG:42 | 201 |  |
| TNKS_Exon8 | rs7006985 | Thr462= | ref=A |  | 111 |  |
| TNKS_Exon8 | rs7006985 | Thr462= | ref=A | TH_418198001_C_AG:42 | 108 |  |
| TNKS_Exon8 | rs7006985 | Thr462= | ref=A | TH_418300001_C_AG:48 | 365 |  |
| TNKS_Exon8 | rs7006985 | Thr462= | ref=A | TH_418302001_C_AG:36 | 141 |  |
| TNKS_Exon8 | rs7006985 | Thr462= | ref=A | TH_418304001_C_AG:41 | 302 |  |
| TNKS_Exon8 | rs7006985 | Thr462= | ref=A |  | 129 |  |
| TNKS_Exon8 | rs7006985 | Thr462= | ref=A |  | 117 |  |
| TNKS_Exon8 | rs7006985 | Thr462= | ref=A | TH_418360001_C_AG:36 | 228 |  |
| TNKS_Exon8 | rs7006985 | Thr462= | ref=A | TH_418361001_C_AG:50 | 208 |  |
| TNKS_Exon8 | rs7006985 | Thr462= | ref=A |  | 190 |  |
| TNKS_Exon8 | rs7006985 | Thr462= | ref=A | TH_418692001_C_AG:31 | 106 |  |
| TNKS_Exon8 | rs7006985 | Thr462= | ref=A |  | 178 |  |
| TNKS_Exon8 | rs7006985 | Thr462= | ref=A |  | 135 |  |
| TNKS_Exon8 | rs7006985 | Thr462= | ref=A |  | 141 |  |
| TNKS_Exon8 | rs7006985 | Thr462= | ref=A | TH_418748001_C_AG:43 | 345 |  |
| TNKS_Exon8 | rs33944167 | Pro478= | ref=G | hetero=TH_1315304001_P_AG:47 | score=268 |  |
| TNKS_Exon8 | rs33944167 | Pro478= | ref=G | TH_1715064001_P_AG:43 | 203 |  |
| TNKS_Exon8 | rs33944167 | Pro478= | ref=G | TH_1715093001_P_AG:50 | 103 |  |
| TNKS_Exon8 | rs33944167 | Pro478= | ref=G | TH_1815241001_P_AG:44 | 270 |  |
| TNKS_Exon8 | rs33944167 | Pro478= | ref=G | TH_1815261001_P_AG:48 | 278 |  |
| TNKS_Exon8 | rs33944167 | Pro478= | ref=G | TH_1815272001_P_AG:41 | 177 |  |
| TNKS_Exon8 | rs33944167 | Pro478= | ref=G | TH_1815285001_P_AG:34 | 228 |  |
| TNKS_Exon8 | rs33944167 | Pro478= | ref=G | TH_1815337001_P_AG:46 | 238 |  |
| TNKS_Exon8 | rs33944167 | Pro478= | ref=G | TH_1815484001_P_AG:44 | 278 |  |
| TNKS_Exon8 | rs33944167 | Pro478= | ref=G | TH_515078001_P_AG:42 | 214 |  |
| TNKS_Exon8 | rs33944167 | Pro478= | ref=G | TH_515091001_P_AG:41 | 258 |  |
| TNKS_Exon8 | rs33944167 | Pro478= | ref=G | TH_515164001_P_AG:48 | 311 |  |
| TNKS_Exon8 | rs33944167 | Pro478= | ref=G | TH_515175001_P_AG:47 | 171 |  |
| TNKS_Exon8 | rs33944167 | Pro478= | ref=G | TH_715088001_P_AG:47 | 149 |  |
| TNKS_Exon8 | rs33944167 | Pro478= | ref=G | TH_4018023001_C_AG:44 | 265 |  |
| TNKS_Exon8 | rs33944167 | Pro478= | ref=G | TH_4018051001_C_AG:42 | 266 |  |
| TNKS_Exon8 | rs33944167 | Pro478= | ref=G | TH_418123001_C_AG:47 | 270 |  |
| TNKS_Exon8 | rs33944167 | Pro478= | ref=G | TH_418124001_C_AG:47 | 272 |  |
| TNKS_Exon8 | rs33944167 | Pro478= | ref=G | TH_418135001_C_AG:33 | 138 |  |
| TNKS_Exon8 | rs33944167 | Pro478= | ref=G | TH_418136001_C_AG:33 | 134 |  |
| TNKS_Exon8 | rs33944167 | Pro478= | ref=G | TH_418148001_C_AG:32 | 238 |  |
| TNKS_Exon8 | rs33944167 | Pro478= | ref=G | TH_418163001_C_AG:48 | 218 |  |
| TNKS_Exon8 | rs33944167 | Pro478= | ref=G | TH_418193001_C_AG:47 | 149 |  |
| TNKS_Exon8 | rs33944167 | Pro478= | ref=G | TH_418360001_C_AG:40 | 210 |  |
| TNKS_Exon8 | rs33944167 | Pro478= | ref=G | TH_418692001_C_AG:40 | 121 |  |

All variants in the exonic regions of the screened genes *FTO*, *TMEM18*, *SDCCAG8*, *TKNS*, *MC4R*, *MSRA* and *TBC1D1* in 196 extremely obese children and adolescents and 176 lean adults. Every deviant call from wild type is listed in one line including the probability of heterozygousity (column “Zygosity”) and the score for the overall validity of the variant (column “Score”).
